# Ultrasonic bath synthesized MIL-88B(Fe)/AgCl/Ag composite for visible-light photocatalytic degradation of anthracene

**DOI:** 10.1038/s41598-026-45178-w

**Published:** 2026-05-06

**Authors:** Pasu Inphak, Prakasit Intaphong, Sujitra Tandorn, Gobwute Rujijanagul, Chamnan Randorn

**Affiliations:** 1 Department of Chemistry, Faculty of Science, Chiang Mai University, Chiang Mai, 50200 Thailand; 2 Office of Research Administration, Chiang Mai University, Chiang Mai, 50200 Thailand; 3 Department of Physics and Materials Science, Faculty of Science, Chiang Mai University, Chiang Mai, 50200 Thailand; 4 Center of Excellence in Materials Science and Technology, Chiang Mai University, Chiang Mai, 50200 Thailand

**Keywords:** Degradation of anthracene, MIL-88B(Fe)/AgCl/Ag, PM2.5, Photocatalyst, Chemistry, Environmental sciences, Materials science

## Abstract

Polycyclic aromatic hydrocarbons (PAHs) are hazardous organic pollutants commonly detected in fine particulate matter (PM2.5), posing significant risks to environmental and human health. In this study, a visible light active composite photocatalyst, MIL-88B(Fe)/AgCl/Ag, was synthesized via a precipitation method followed by photoreduction to enhance the photocatalytic performance of MIL-88B(Fe) for anthracene degradation. The ternary composite exhibited significantly improved photocatalytic activity compared with pristine MIL-88B(Fe) and AgCl/Ag under visible-light irradiation, which is attributed to the formation of an efficient heterojunction that promotes charge separation and reactive species generation. Scavenger experiments and electron paramagnetic resonance (EPR) spectroscopy revealed that singlet oxygen (^1^O_2_) is the dominant reactive species, while hydroxyl radicals (^•^OH) play a secondary role in the degradation process. Product analysis using UV–vis spectroscopy and GC–MS confirmed that anthracene is initially oxidized to 9,10-anthraquinone through ^1^O_2_ and ^•^OH. Further photochemical reactions lead to smaller aromatic products such as benzaldehyde, benzyl alcohol, and their methylated derivatives. The synergistic interaction between Ag/AgCl and MIL-88B(Fe) enables dual oxidation pathways and promotes charge separation, resulting in enhanced photocatalytic efficiency. These findings provide insight into the photocatalytic degradation pathways of anthracene and suggest the potential of MOF-based composite photocatalysts for visible-light-driven environmental remediation.

**Supplementary Information:**

The online version contains supplementary material available at 10.1038/s41598-026-45178-w.

## Introduction

Air pollution remains a critical environmental challenge worldwide, with fine particulate matter (PM2.5) recognized as one of the most harmful pollutants affecting human health^1^. Due to their small size, PM2.5 particles can penetrate deeply into the respiratory system and bloodstream, contributing to respiratory and cardiovascular diseases as well as premature mortality^2,3^. A substantial fraction of PM2.5 consists of organic carbon containing polycyclic aromatic hydrocarbons (PAHs), a group of toxic, mutagenic, and carcinogenic compounds generated mainly from incomplete combustion processes^4^. Common PAHs detected in PM2.5 include phenanthrene, anthracene, fluoranthene, pyrene, benzo[a]anthracene, and chrysene^5–7^. These compounds contain fused aromatic rings with delocalized π-electrons, giving them high chemical stability and environmental persistence^8^. After emission, PAHs associated with PM2.5 may deposit onto environmental surfaces or partition into aqueous media such as atmospheric moisture, surface water, or wastewater streams. Consequently, aqueous-phase systems are frequently employed as simplified model environments to investigate PAH degradation mechanisms under controlled conditions. Among PAHs, anthracene is widely used as a model compound for degradation studies because of its well-defined aromatic structure and representative reactivity^9^.

Therefore, developing effective technologies for the degradation of PAHs such as anthracene has become an important research objective. Various technologies have been explored for the degradation of organic pollutants, including thermochemical and biological processes, photocatalysis, molecular oxygen activation (MOA) technology^10^, and persulfate-based advanced oxidation process^11^. Of these approaches, photocatalysis has attracted increasing attention because it utilizes solar or artificial light energy to drive pollutant degradation in a sustainable and environmentally friendly manner^12,13^.

Several reactive oxygen species generated in photocatalytic systems have been reported to contribute to anthracene degradation. Hydroxyl radicals (•OH)^14,15^, superoxide radicals (^•^O_2_⁻)^16^, and singlet oxygen (^1^O_2_)^17–19^ are commonly involved in photocatalytic oxidation of aromatic compounds. However, photocatalytic efficiency strongly depends on factors such as light-harvesting capability, charge separation efficiency, active-site availability, and adsorption capacity^20,21^. Improving the adsorption of hydrophobic PAHs on catalyst surfaces is particularly important for initiating photocatalytic reactions. High-surface-area materials have therefore been incorporated into photocatalysts to enhance pollutant adsorption, including chitosan-supported TiO_2_^22^ or coconut fiber-AgTiO_2_ composites^23^. In this context, metal–organic frameworks (MOFs) have emerged as promising photocatalytic platforms owing to their exceptionally high surface area, tunable porosity, and structural versatility. Constructed from metal clusters coordinated with organic ligands^24,25^, MOFs exhibit exceptional properties for applications such as gas sorption, sensors, drug delivery, heterogeneous catalysis and energy-related processes^26–30^. Despite these advantages, the application of MOF-based materials for photocatalytic degradation of PAHs such as anthracene remains relatively unexplored^31,32^.

Fe-based MOFs are particularly attractive candidates because of the abundance of iron, visible-light responsiveness, and the presence of Fe–O clusters capable of participating in photocatalytic redox reactions^33^. Supporting this notion, iron oxides such as goethite (α-FeOOH) and magnetite (Fe_3_O_4_) have been reported to catalyze anthracene degradation under UV light^34^, suggesting that analogous Fe–O clusters in Fe-MOFs could enable similar photocatalytic activity. MIL-88B(Fe) stands out as a synthetically accessible and robust MOF with tunable porosity and strong visible-light absorption^21^, making it a promising platform for developing composite photocatalysts aimed at selective PAH degradation^35^. However, rapid recombination of photogenerated charge carriers remains a major limitation for many photocatalysts^36^. This issue can be addressed by constructing composite photocatalysts that enhance charge mobility and suppress recombination^37,38^. In particular, AgCl/Ag coupled with Fe-based metal–organic frameworks (MOFs) have emerged as a promising candidate due to its effective charge transfer capabilities and facile synthesis^39,40^.

In this study, a MIL-88B(Fe)/AgCl/Ag composite photocatalyst was designed and synthesized for visible-light-driven degradation of anthracene. The incorporation of AgCl/Ag into the MIL-88B(Fe) framework is expected to enhance charge separation and promote the generation of reactive oxygen species. In addition to evaluating photocatalytic performance, the degradation pathway of anthracene was investigated through reactive-species trapping experiments and product analysis, enabling tentative identification of key intermediates and transformation routes involved in the photocatalytic process.

## Experiment

### Materials

All chemicals used were of analytical grade. FeCl_3_•6 H₂O was sourced from QReC, while terephthalic acid (H_2_BDC) and acrylamide were acquired from Sigma-Aldrich. Dimethylformamide (DMF), absolute ethanol (CH₃CH₂OH), and isopropanol (CH_3_CH(CH_3_)OH) were obtained from ACI Labscan. Silver nitrate (AgNO_3_) and sodium chloride (NaCl) were procured from QReC. Rhodamine B was supplied by LOBA CHEMIE PVT. LTD., while dichloromethane and acetone were purchased from ACI Labscan. Anthracene was acquired from Sigma-Aldrich.

### Synthesis of MIL-88B(Fe)

The iron-based MIL-88B material was synthesized using a commercially available ultrasonic bath, which provides mild ultrasonic agitation at low cost and without specialized equipment^41^. A mixture comprising FeCl_3_•6H_2_O (7.5 mmol, 2.027 g) and H_2_BDC (7.5 mmol, 1.246 g) dissolved in 30 mL of dimethylformamide (DMF) was subjected to sonication in a low-intensity ultrasonic bath at 80 °C for 2 h. Following the cooling of the solution, orange precipitates were isolated from the DMF solvent via centrifugation and subsequently washed thrice with DMF, deionized (DI) water, and ethanol. The resulting powders were then dried at 120 °C for 24 h. The product was labeled as M88.

### Synthesis of MIL-88B(Fe)/AgCl/Ag composite

MIL-88B(Fe)/AgCl/Ag composites were synthesized via in situ precipitation of AgCl onto MIL-88B(Fe), followed by photoreduction under visible light, adapting a previously reported method^42^. MIL-88B(Fe) (1.0 g) was dispersed in 100 mL of AgNO_3_ solution, with varying AgNO_3_ amounts (0.3, 0.5, and 1.0 g, corresponding to 1.77, 2.94, and 5.88 mmol, respectively). The mixture was stirred for 30 min to facilitate Ag⁺ ion adsorption onto the MIL-88B(Fe) surface. Subsequently, 100 mL of 0.2 M NaCl was added to precipitate Ag⁺ ions as AgCl, forming MIL-88B(Fe)/AgCl (M88-AC).

The mixture was then irradiated under a 50 W warm white LED lamp (500–700 nm) for 2 h to reduce Ag⁺ to Ag^0^, forming MIL-88B(Fe)/AgCl/Ag (M88-AG). Products synthesized with varying AgNO_3_ amounts were labeled as M88-AGX.X (X.X = 0.3, 0.5, 1.0). Pristine AgCl/Ag was also synthesized using a similar method for comparison.

All photocatalysts were characterized using powder X-ray diffraction (PXRD, RIGAKU SMARTLAB), X-ray photoelectron spectroscopy (XPS, AXIS Ultra DLD), scanning electron microscopy with energy-dispersive X-ray spectroscopy (SEM-EDS, JSM IT800), and transmission electron microscopy (TEM, JEM 2100 Plus). Electrochemical impedance spectroscopy (EIS) measurements were performed using AUTOLAB PGSTAT320N 2500.

### Photocatalytic activity determination

To evaluate photocatalytic activity and optimize the AgCl/Ag composition, the degradation of Rhodamine B (RhB) was employed as a preliminary model reaction. A relatively high concentration of RhB (200 mg/L) was used to rigorously test the catalytic performance. The catalyst (0.025 g) was dispersed in 25 mL of the dye solution, and adsorption equilibrium was established by stirring the suspension in the dark for 12 h at a stirring speed of 100 rpm. The mixture, contained in a glass beaker, was then irradiated under visible light using a 50 W LED lamp (500–700 nm) positioned 2 cm from the reactor. The light intensity at the reactor surface was measured using a commercial lux meter and was approximately 1,700 lx, corresponding to an estimated irradiance of 1.7 mW/cm^2^). During the reaction, aliquots were withdrawn at designated time intervals and immediately centrifuged to remove the catalyst particles, thereby quenching the reaction. The residual RhB concentration was subsequently determined by UV–vis spectroscopy at the characteristic absorption wavelength of 554 nm.

For the degradation of anthracene, a 40 mg/L solution of anthracene (prepared in a Acetone: DI-water mixture, 7:3 by volume) was stirred with M88, M88-AG and Ag/AgCl catalysts in closed-system using screw cap flask for 12 h to achieve adsorption equilibrium. The suspensions were then irradiated with a 50 W LED lamp like the previous experiment. At designated intervals, 25 mL samples were withdrawn for analysis. Anthracene concentrations were determined using UV–visible spectroscopy in scan mode. To identify degradation products, the reaction mixtures were extracted with dichloromethane (CH_2_Cl_2_) 10 mL, concentrated to a final volume of 2 mL, and analyzed quantitatively by gas chromatography–mass spectrometry (GC-MS).

Reactive species involved in the photocatalytic process were identified using specific scavengers: tert-Butyl alcohol (TBA) 20 mM for hydroxyl radicals (^•^OH), p-benzoquinone (BQ) 1 mM for superoxide anion radicals (^•^O_2_^−^), ammonium oxalate (AO) 10 mM for hole (h^+^), silver nitrate (AgNO_3_) 5 mM for electron (e^−^) and sodium azide (NaN_3_) 10 mM for singlet oxygen (^1^O_2_)^43^. Each scavenger was introduced into the reaction system with M88-AG prior to light irradiation, and the photocatalytic efficiency was observed using UV–visible spectroscopy in scan mode.

The generation of radical species was further confirmed by electron paramagnetic resonance (EPR) spectroscopy using 5,5-Dimethyl-1-pyrroline N-oxide (DMPO) as a spin-trapping agent. In a typical procedure, 8 mg of catalyst was dispersed in 2 mL of an acetone–water solvent, followed by the addition of 0.8 mL of DMPO solution (40 mg DMPO in 10 mL solvent). The suspensions were exposed to the same LED light source used in the photocatalytic experiments for 1 min. Immediately after irradiation, the sample was subjected to EPR analysis to detect the characteristic signals of trapped radical species.

## Results and discussions

### Characterizations

**Fig. 1:**
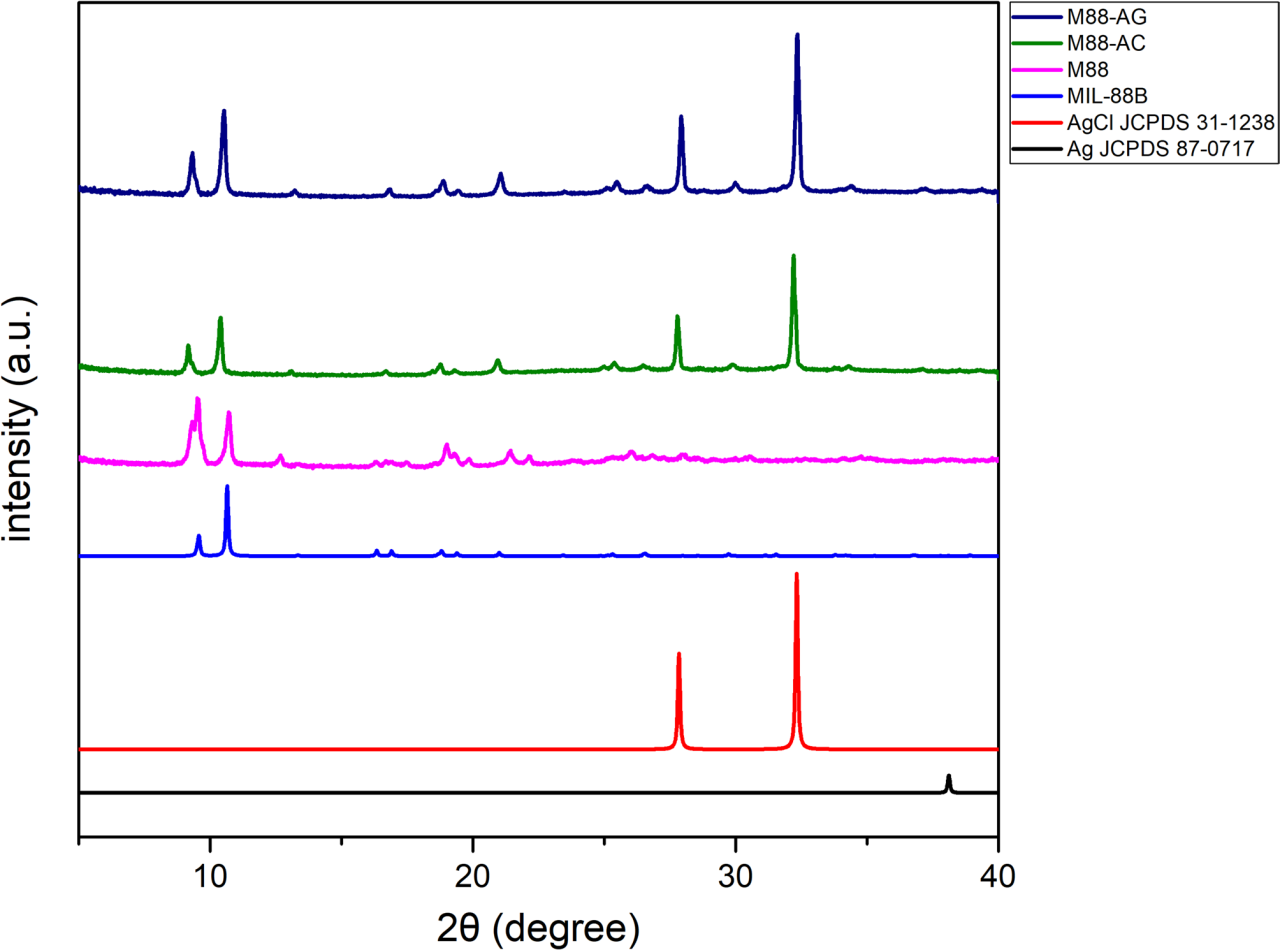
XRD pattern of M88, M88-AC and M88-AG.

The XRD patterns of the catalysts are shown in Fig. 1. MIL-88B(Fe) exhibits diffraction peaks at 9.45°, 10.27°, 16.21°, 16.66°, 18.87°, and 19.36°, which are consistent with the simulated pattern of MIL-88B(Fe) and previously reported data^44^. These peaks confirm the formation of the desired crystalline phase. However, slight deviations in peak intensities, particularly the enhanced intensity at 9.45°, suggest the presence of structural irregularities or guest species within the MOF cavities^44^. Interestingly, upon incorporation into the AgCl/Ag composite (M88-AG), these unusual features diminish, indicating that the composite synthesis process may help eliminate or reduce such impurities, possibly by displacing trapped molecules or restructuring the MOF framework.

The XRD patterns of M88-AC and M88-AG show diffraction peaks corresponding to both MIL-88B(Fe) and AgCl (JCPDS No. 31–1238), confirming the successful incorporation of these phases into the composite. In contrast, the characteristic peak of metallic Ag (JCPDS No. 87–0717) is barely detectable, likely due to its low crystallinity, limited content, or nanoscale dispersion, all of which contribute to weak diffraction intensity^45,46^. Furthermore, the possible random distribution of Ag within the AgCl lattice may obscure its distinct XRD signature. To confirm the presence of metallic Ag, XPS analysis was conducted. Notably, a similar trend was observed in the AgCl/Ag sample synthesized without MIL-88B(Fe), where only weak Ag peaks appeared in the XRD pattern as shown in Figure S1 (Supporting Information). Nonetheless, both AgCl and a minor Ag phase were confirmed by complementary XPS and EDS analyses.

**Fig. 2:**
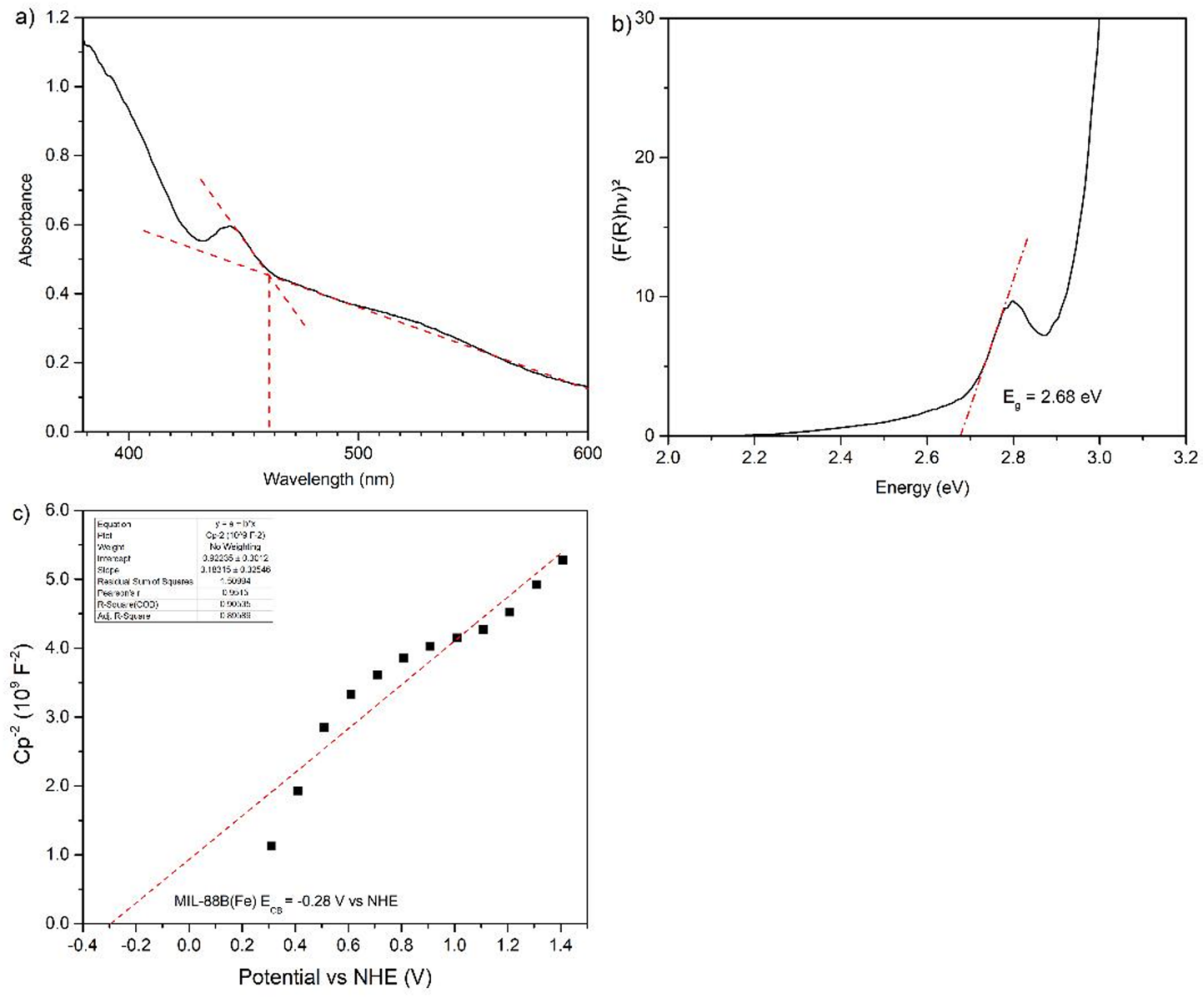
(**a**) UV-vis DRS spectrum of M88 (**b**) Tauc plot of M88 and (**c**) Mott-Schottky plot of M88.

The UV-vis diffuse reflectance (DRS) spectra of M88 (Fig. 2a) exhibited an absorption edge onset at 467 nm, consistent with the orange coloration of the powder. This observation implies effective excitation of valence band electrons by visible light, a pivotal characteristic for photocatalytic processes. The band gap energy was estimated using the Kubelka–Munk function derived from the diffuse reflectance spectra. As shown in Fig. 2b, the Tauc plot was constructed by plotting (F(R)ℎ)^2^ versus photon energy (ℎ), assuming a direct electronic transition (*n* = 2)^47^, which is commonly applied for Fe-based MOF photocatalysts^47^. From the extrapolation of the linear region, the band gap energy of M88 was determined to be 2.68 eV, consistent with the absorption edge observed in the UV–Vis DRS spectrum.

The Mott-Schottky plot of M88 (Fig. 2c) was analyzed to determine the semiconductor’s band energy. The positive slope of the linear trend line indicates an N-type semiconductor, with the x-intersection providing the potential energy of the conduction band (E_CB_). The E_CB_ of M88 was calculated to be −0.28 eV (vs. NHE), Furthermore, the potential energy of the valence band (E_VB_) was determined to be + 2.41 eV, derived from the difference between E_CB_ and E_g_. This band energy of MIL-88B(Fe) relates with previous research^48^.

**Fig. 3:**
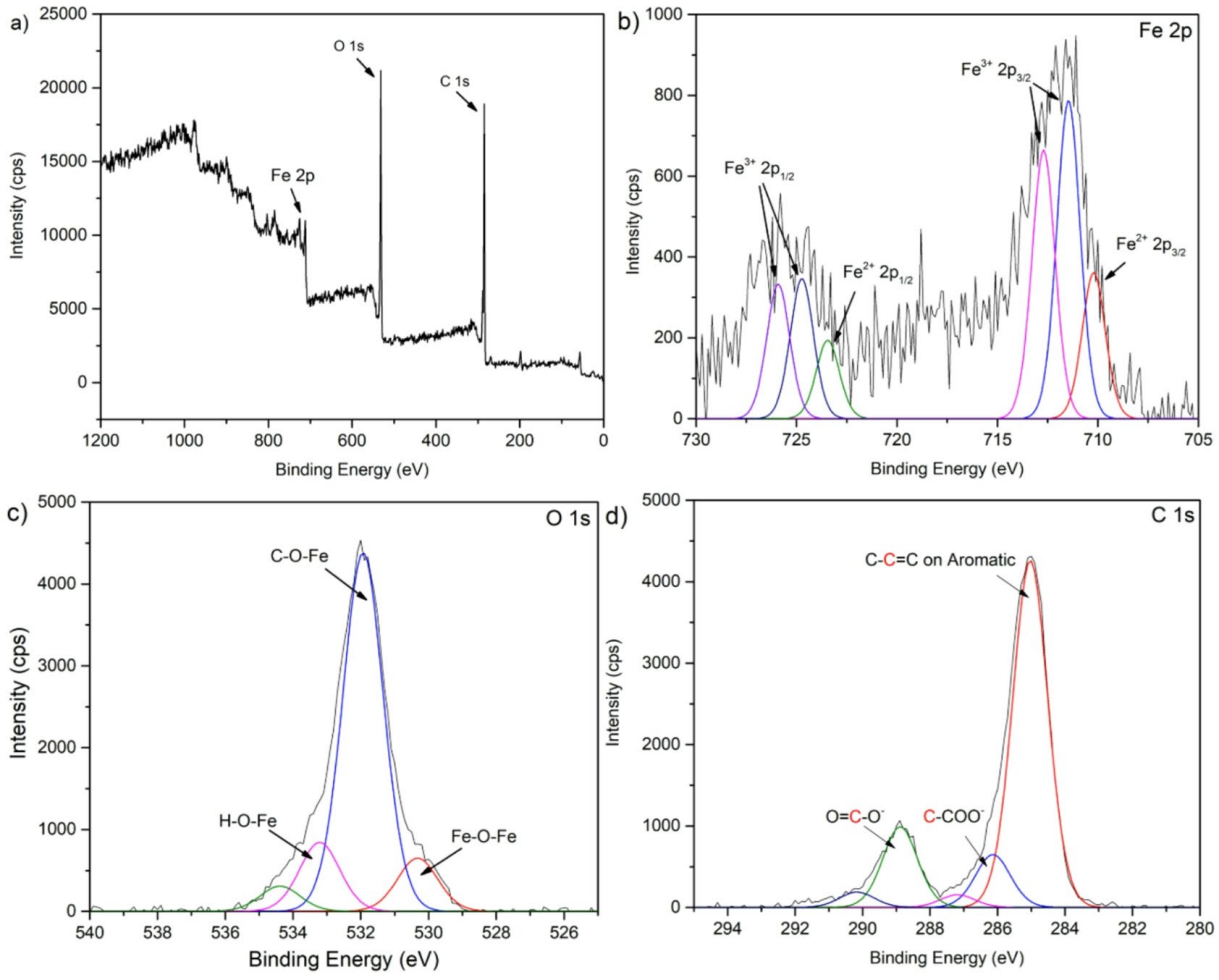
XPS spectra of M88 (**a**) survey scan (**b**) Fe-2p spectra (**c**) O-1s spectra and (**d**) C-1s spectra.

The XPS spectra of M88 are shown in Fig. 3. The wide-scan spectrum (Fig. 3a) reveals the presence of Fe 2p, O 1 s, C 1 s, and Cl 2p, confirming the elemental composition of the sample. High-resolution spectra for Fe 2p, O 1 s, and C 1 s are presented in Figs. 3b–d, respectively. In the Fe 2p spectrum (Fig. 3b), the peaks at 711.5 eV and 724.7 eV are assigned to Fe^3+^ 2p_3/2_ and Fe^3+^ 2p_1/2_, respectively, along with satellite peaks at 712.7 eV and 725.9 eV, which are characteristic of the Fe³⁺ oxidation state. Additionally, the peaks at 710.2 eV and 723.4 eV are attributed to Fe²⁺ 2p_3/2_ and 2p_1/2_ indicating the coexistence of both Fe^2+^ and Fe^3+^ within the MIL-88B(Fe) structure^49^. These two oxidation states of iron are intermixed within the metal cluster of the MIL-88B(Fe) structure, allowing for interchange between Fe^3+^ and Fe^2+^ during photocatalytic reactions. The O 1 s emission spectrum can be deconvoluted into three principal peaks: the peak at 530.3 eV is attributed to Fe–O–Fe bonds, typically associated with lattice oxygen coordinated to Fe^3+^; the peak at 531.9 eV corresponds to C–O–Fe linkages within the organic ligand framework; and the component at 533.2 eV is assigned to surface hydroxyl groups (H–O–Fe), indicating adsorbed water or hydroxyl species^50^. This substantiates the bonding between the metal and the ligand within the structure. Furthermore, distinct signals corresponding to carbon in various chemical environments are evident in the C 1 s spectra: 285.0 eV for carbon within aromatic rings, 286.1 eV for carbon adjacent to the carboxylic group, and 288.9 eV for carbon within the carboxylic group^50^. This observation confirms the presence of the organic linker, terephthalate, within the structure^51^.

**Fig. 4:**
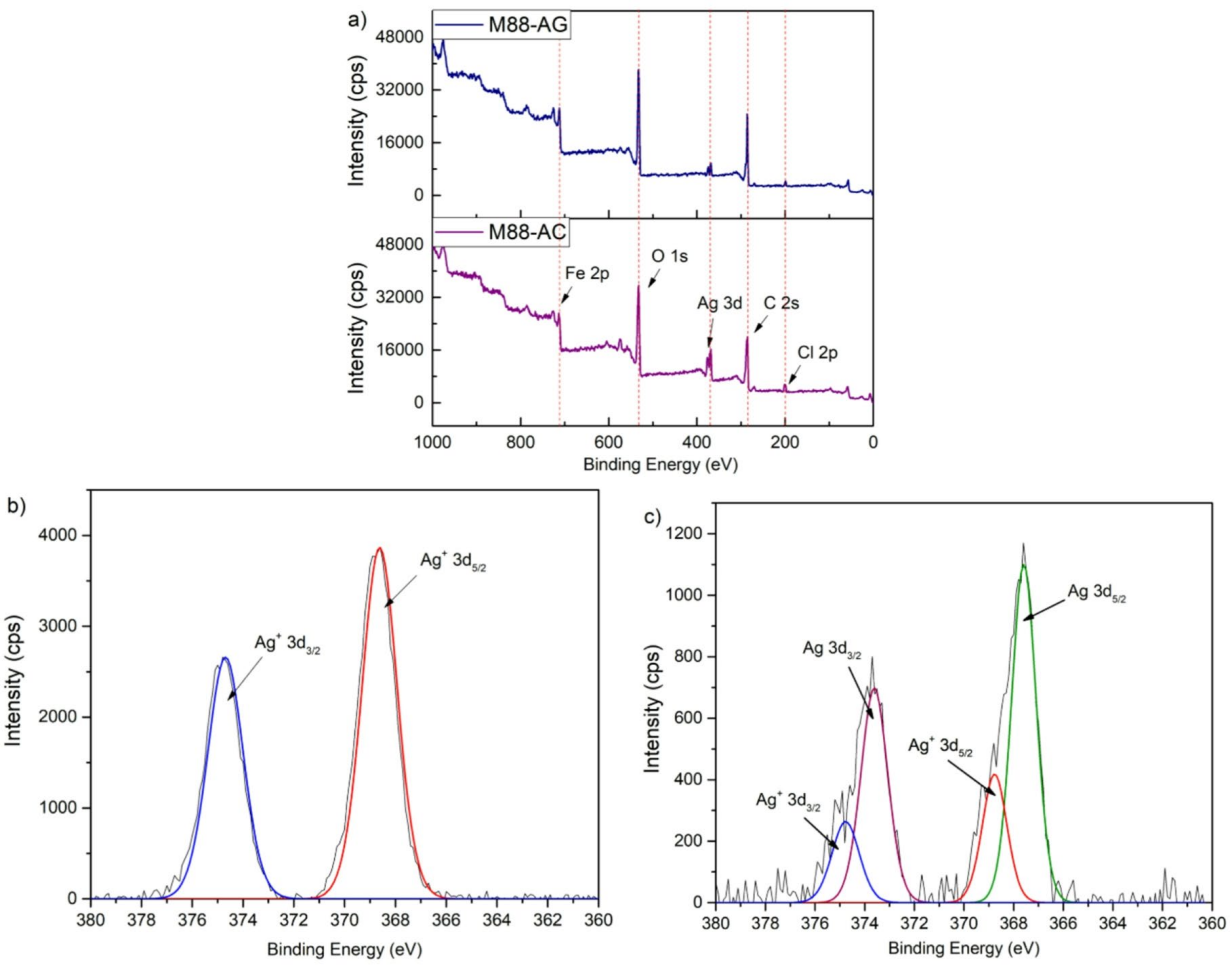
XPS spectra (**a**) survey scan of M88-AG and M88-AC and (**b**) Ag spectra of M88-AC (before photoreduction) (**c**) Ag spectra of M88-AG (after photoreduction).

The XPS spectra of M88-AG and M88-AC are shown in Fig. 4. The wide-scan spectra (Fig. 4a) confirm the presence of all expected elements in the composites. In the high-resolution Ag 3 d spectrum (Fig. 4c), the Ag signals can be deconvoluted into two sets of peaks, corresponding to different oxidation states. The peaks at 368.7 eV (Ag 3d_5/2_) and 374.8 eV (Ag 3d_3/2_) are attributed to Ag⁺, indicating the presence of AgCl. Meanwhile, the peaks at 367.6 eV and 373.6 eV correspond to metallic silver (Ag⁰), confirming the partial photoreduction of Ag⁺ to Ag⁰ during synthesis^45^. Comparatively, the high-resolution Ag spectra in Fig. 4b for M88-AC exhibit peaks solely corresponding to Ag^+^ at 368.7 eV and 374.8 eV. These findings confirm the photoreduction of Ag^+^ into Ag within the composite following light irradiation^42^.

**Fig. 5:**
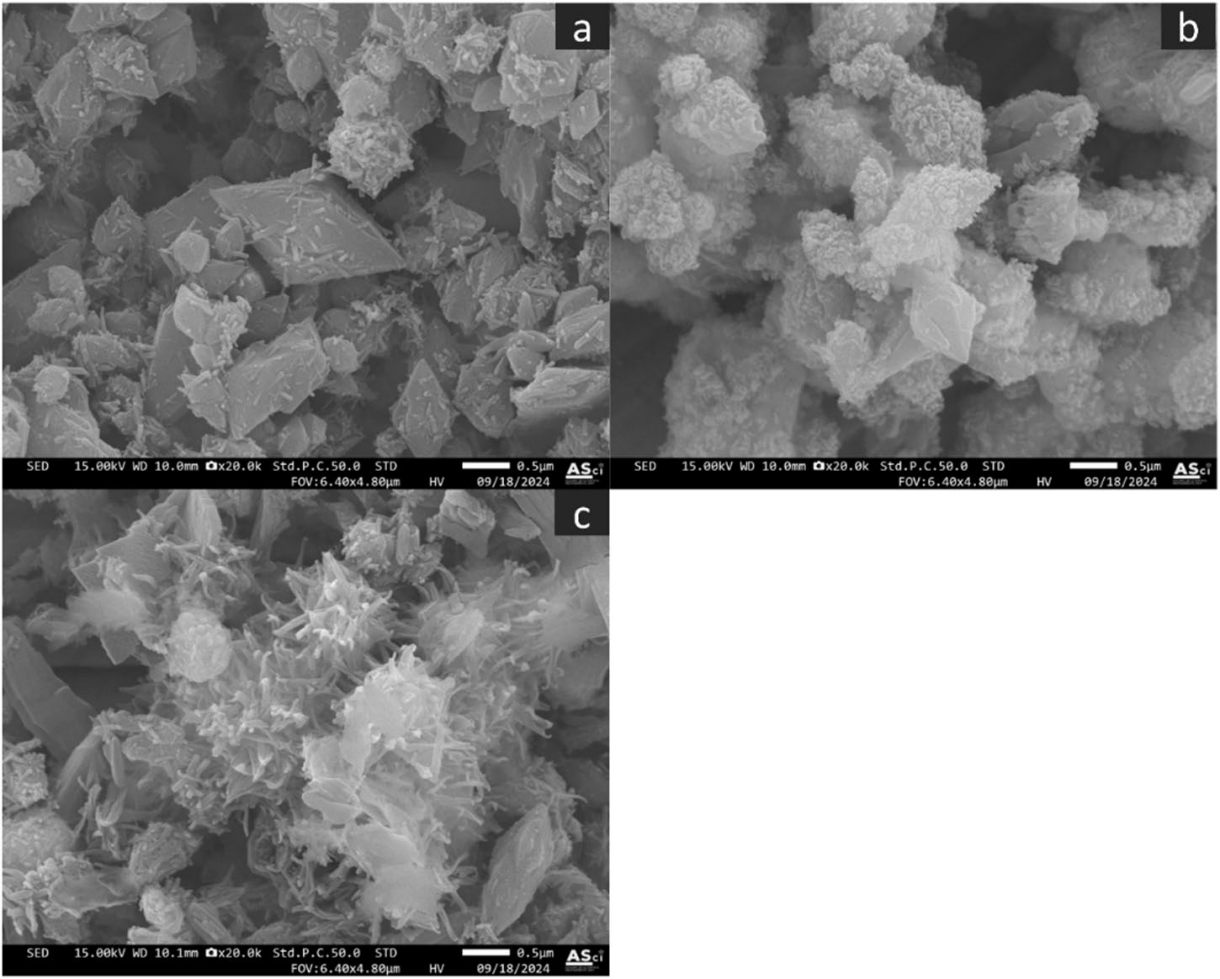
SEM images X20,000 (**a**) M88 (**b**) M88-AC (**c**) M88-AG.

The morphology of the synthesized catalysts was investigated using scanning electron microscopy (SEM), as shown in Fig. 5. The M88 sample (Fig. 5a) exhibits an octahedral crystal structure, consistent with the previously reported morphology of MIL-88B(Fe)^50,51^. Upon incorporating AgCl into the Fe-MOF framework, the resulting M88-AC composite (Fig. 5b) displays the formation of partially rounded AgCl crystals on the surface of the MOF^52^. Following visible light irradiation, a distinct morphological transition is observed, where AgCl crystals evolve into flower-like rod structures. This transformation is indicative of the photoinduced reduction of AgCl to metallic Ag, which occurs under light exposure, confirming the dynamic nature of the Ag/AgCl phase transition.

Further evidence for the successful dispersion of Ag and AgCl phases is provided by elemental mapping via energy-dispersive X-ray spectroscopy (EDS) (Figure S2). The EDS mapping shows a uniform distribution of Ag and Cl elements across the MOF matrix in the M88-AG composite. Quantitative EDS analysis confirms that the elemental composition of M88 is in close agreement with the theoretical formula of MIL-88B(Fe), Fe_3_O(OOCC_6_H_4_COO)_3_Cl^51^. Moreover, a decrease in the Cl content accompanied by an increase in the Ag content from M88-AC to M88-AG supports the photoreduction of Ag⁺ to metallic Ag. This compositional shift confirms the successful transformation of AgCl/MIL-88B(Fe) into AgCl/Ag/MIL-88B(Fe) under visible light irradiation. Table 1.

**Table 1:** Elemental analysis by EDS of M88, M88-AC and M88-AG.

elements	%Atomic				
	Fe	C	O	Cl	Ag
M88	6.57	67.56	21.54	4.33	0.00
M88-AC	1.93	48.70	23.69	7.41	18.27
M88-AG	2.12	45.91	16.45	2.12	25.96

**Fig. 6:**
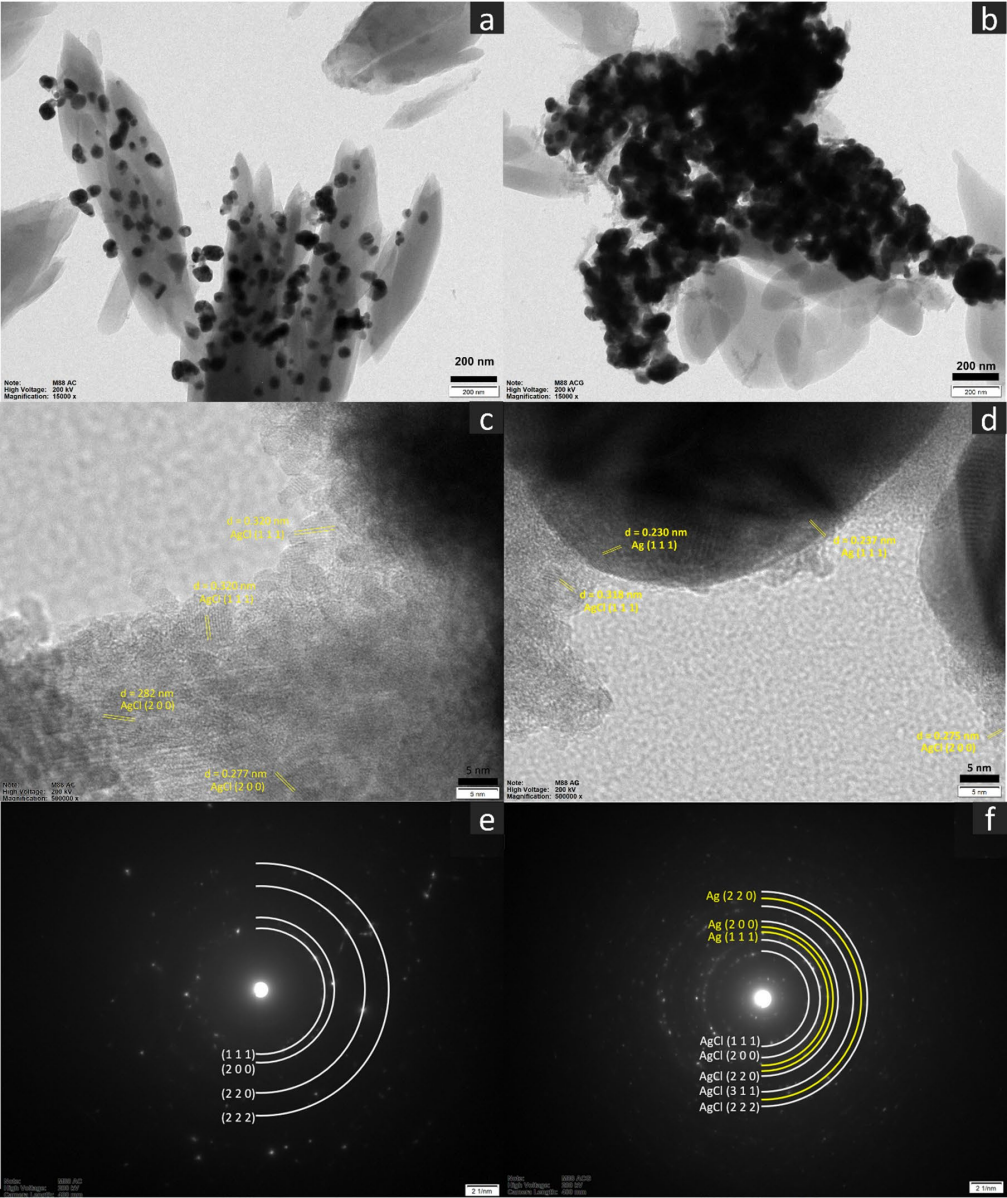
TEM images (**a**) M88-AC, (**b**) M88-AG and HR-TEM images (**c**) M88-AC, (**d**) M88-AG and SAED pattern (**e**) M88-AC, (**f**) M88-AG.

The presence of the Ag phase in M88-AG was further confirmed through TEM analysis, as illustrated in Fig. 6. Figure 6a and b demonstrate AgCl and Ag/AgCl phases on MIL-88B(Fe), respectively, with the high atomic weight phases appearing as dark regions and MIL-88B(Fe) as bright regions. High-resolution TEM (HR-TEM) images (Fig. 6c and d) reveal the crystalline nature of AgCl and Ag, displaying distinct lattice spacing of atomic planes. Additionally, selected area electron diffraction (SAED) patterns, shown in Fig. 6e and f, were generated. These ring patterns reveal that M88-AG exhibits diffraction patterns corresponding to both AgCl and Ag phases, whereas M88-AC only displays patterns associated with AgCl.

### Photocatalytic dyes degradation activity

To assess the influence of the Ag/AgCl ratio in MOF-based photocatalysts, the photocatalytic degradation of Rhodamine B (RhB) under visible light was investigated using M88 and a series of Ag/AgCl composites denoted as M88-AGX.X. After overnight adsorption, the M88 sample exhibited a higher dye adsorption capacity than its composites. This is likely due to the porous surface of the MOF being partially blocked by the Ag/AgCl phase, which forms during composite synthesis, thereby reducing the surface area available for dye interaction.

Despite its superior adsorption, M88 displayed slower photocatalytic activity compared to its composites. As shown in Figure S3a, the M88-AG series exhibited significantly enhanced RhB degradation, which can be attributed to the heterojunction structure between Ag, AgCl, and MIL-88B(Fe), promoting more efficient charge separation and reduced electron-hole recombination. Among the tested composites, M88-AG0.5 showed the highest photocatalytic efficiency, degrading 96% of 200 mg/L RhB within 10 h. Based on this result, M88-AG0.5 was selected as the optimized composition for further experiments and is henceforth referred to as M88-AG.

Further insight into the degradation mechanisms was obtained from UV–Vis spectral analysis of RhB over time (Figure S4). The spectrum of M88 reveals a shift in the maximum wavelength (λ_max_) from 554 nm to 510 nm, indicating a stepwise de-ethylation process, consistent with previously reported mechanisms^51^. This transformation corresponds to a visible color change from pink to yellow. In contrast, the spectrum of M88-AG showed a steady decrease in absorbance at 554 nm without any shift in λ_max_, suggesting a different degradation pathway, possibly involving direct bond cleavage rather than stepwise de-ethylation. These results imply that M88 and M88-AG utilize distinct reactive species or mechanisms in their photocatalytic processes.

### Photocatalytic anthracene degradation activity

**Fig. 7:**
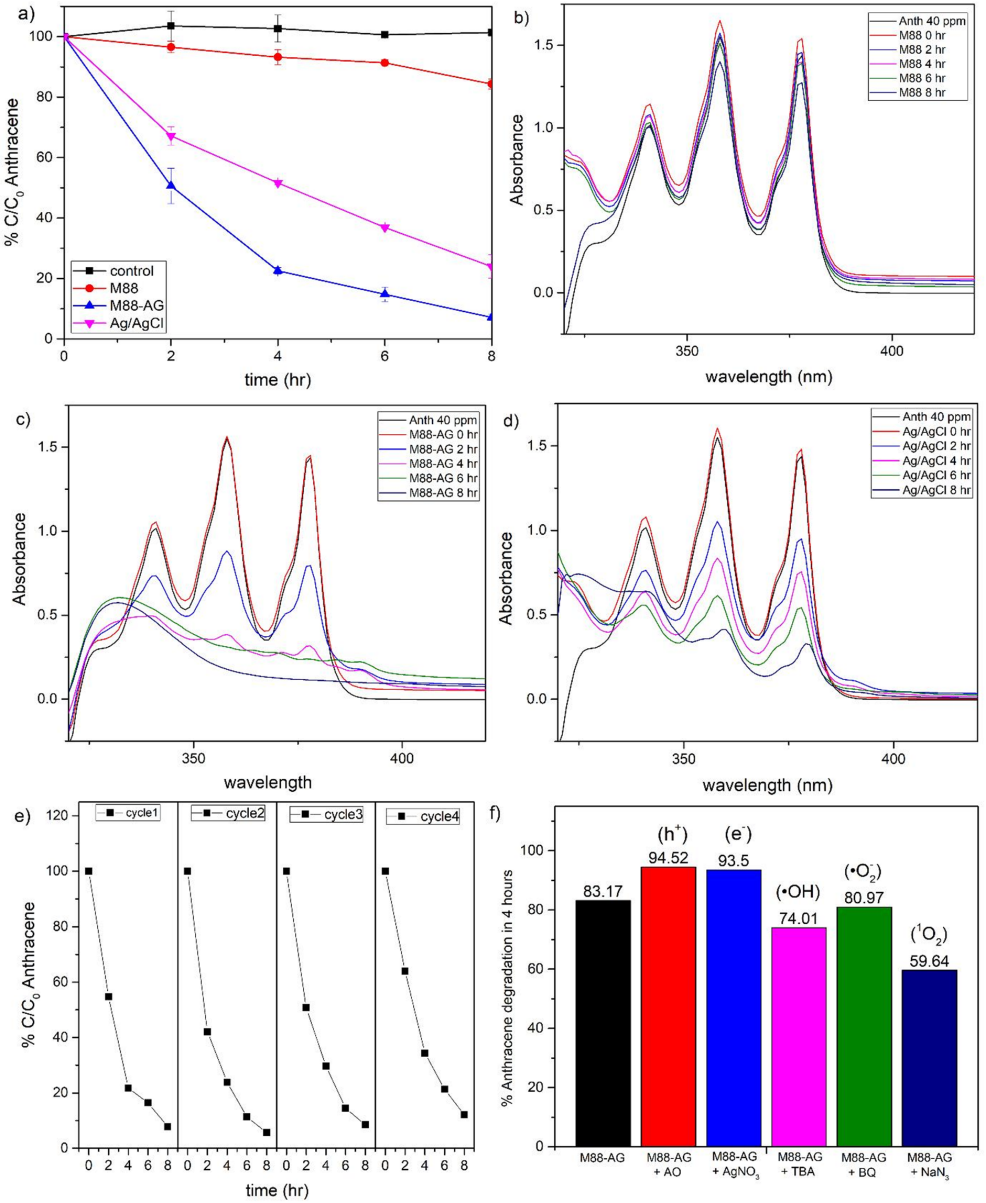
Comparison of photocatalytic performance in anthracene degradation (**a**), UV–Vis spectral changes during the photocatalytic degradation of anthracene using (**b**) M88, (**c**) M88-AG, and (**d**) Ag/AgCl; (**e**) Reusability assessment of M88-AG; (**f**) Effect of scavengers on anthracene degradation efficiency using M88-AG.

In terms of anthracene degradation, Fig. 7a shows that M88-AG achieved the highest degradation rate, as indicated by the decline in anthracene concentration. The degradation followed first-order kinetics, with calculated half-lives of M88 (32.43 h), M88-AG (2.20 h), and Ag/AgCl (3.67 h), as shown in Figure S5. UV-Vis spectroscopy was used to monitor anthracene concentration, and the corresponding spectra are shown in Fig. 7b and d. Anthracene displays characteristic absorption bands at 392, 372, 355, and 338 nm^53^, and the 379 nm peak, slightly red-shifted due to solvent effects, was used for quantification due to its stability against spectral noise^54^.

In the later stages of degradation using M88-AG, the UV-Vis spectrum revealed the appearance of broad absorption bands,, which was not observed for M88 and Ag/AgCl, indicating the formation of intermediate products that may include anthrone or 9,10-anthraquinone. The UV-Vis spectrum of 9,10-anthraquinone, as reported by Khan and Khan, features two main absorption ranges: 275–340 nm and 210–275 nm^55^, supporting its identification as a degradation product. Figure 7e demonstrates the reusability of M88-AG over four photocatalytic cycles, with only a slight reduction in degradation efficiency, indicating good stability and recyclability of the catalyst under the tested conditions.

To elucidate the photocatalytic degradation mechanism of anthracene, specific radical scavengers were employed to identify the active species involved during the reaction. Methanol (MeOH) acts as a hole (h⁺) scavenger, p-benzoquinone (BQ) targets superoxide anion radicals (^•^O_2_⁻), and tert-Butyl alcohol (TBA) is known to quench hydroxyl radicals (^•^OH). In addition, silver nitrate (AgNO_3_) was used as an electron (e⁻) scavenger, while sodium azide (NaN_3_) was applied to quench singlet oxygen (^1^O_2_)^43^.

A comparative photocatalytic performance test was conducted using M88-AG in the presence of different radical scavengers. As shown in Fig. 7f, the addition of NaN_3_ significantly suppressed the anthracene degradation efficiency compared with the control experiment (without scavengers), indicating that singlet oxygen (^1^O_2_) is the dominant reactive species involved in the degradation process. Generally, ^1^O_2_ can be generated through several pathways, such as the reaction between ^•^O₂⁻ and ^•^OH; however, it is more commonly produced via energy transfer from excited photocatalysts to molecular oxygen (triplet oxygen, ^3^O_2_)^56^. Photocatalysts capable of efficiently exciting ^3^O_2_ typically contain highly conductive or single-metal active sites, such as Pt/TiO_2_^17^, rGO-Ag-Cu-Ni^57^, and conductive g-C_3_N_4_ systems^58^, which facilitate rapid electron transfer and energy migration within the structure. In the present system, the generation of ^1^O_2_ is likely associated with the Ag/AgCl phase, where surface plasmon resonance (SPR) can enhance charge separation and electron transfer, thereby promoting the activation of molecular oxygen and the formation of ^1^O_2_^59,60^.

Another scavenger that can slightly exhibit the Anthracene degradation is tert-butyl alcohol (TBA), implying that ^•^OH radicals also participate in the photocatalytic process. These results suggest that ^•^OH radicals act as secondary reactive species contributing to anthracene decomposition. This observation is consistent with previous studies on anthracene photocatalysis^61,62^, where ^•^OH radicals were reported to oxidize anthracene to intermediates such as anthrone and anthraquinone, which are associated with characteristic UV–Vis spectral features. The generation of ^•^OH radicals in this system is likely related to hole (h⁺) mediated oxidation processes occurring on both the MIL-88B(Fe) framework and the AgCl phase. To further verify the formation of these reactive radicals during photocatalysis, electron paramagnetic resonance (EPR) spectroscopy was performed for all catalysts.

**Fig. 8:**
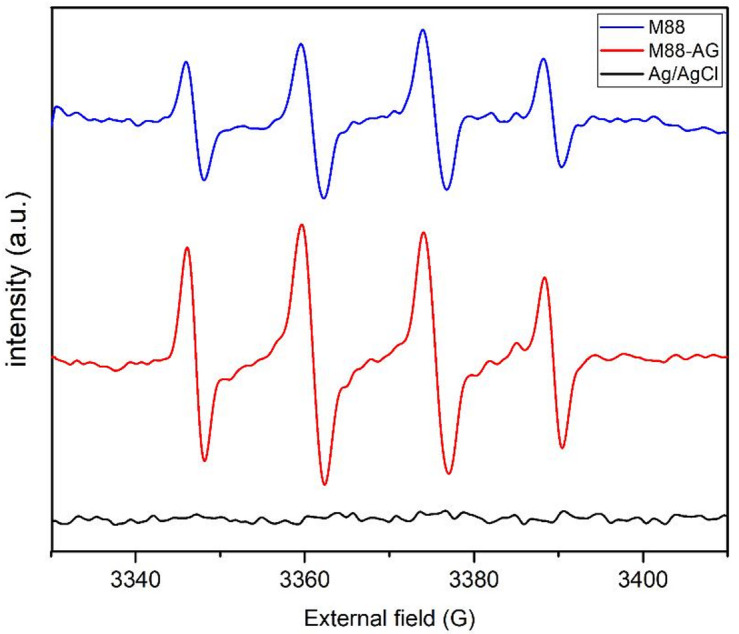
EPR spectra of radical species trapped by DMPO in catalyst suspensions under light irradiation.

The EPR spectra of radical species trapped by DMPO in catalyst suspensions are shown in Fig. 8. Characteristic four-line signals corresponding to the DMPO–^•^OH adduct were clearly observed under light irradiation, confirming the formation of hydroxyl radicals^63^. The M88-AG catalyst exhibited the strongest EPR signal intensity compared with pristine M88, indicating enhanced radical generation in the composite system. In contrast, no detectable radical signals were observed for Ag/AgCl under light irradiation, suggesting that radical formation is not directly driven by this component. Combined with the scavenger experiments, these results suggest that singlet oxygen (^1^O_2_) is the dominant reactive species associated with the Ag/AgCl phase, while ^•^OH radicals are primarily generated from the Fe-MOF (MIL-88B(Fe)) framework.

The residual scavenger tests shown in Fig. 7f reveal several interesting observations. Except for p-benzoquinone (BQ), which does not significantly affect the anthracene degradation rate, the addition of ammonium oxalate (AO) and AgNO_3_ slightly enhances the degradation performance. A plausible explanation is that AgNO_3_ can be photoreduced to metallic Ag on the catalyst surface, which may enhance the surface plasmon resonance (SPR) effect and promote the generation of singlet oxygen (^1^O_2_). Meanwhile, AO can interact with photogenerated holes and the AgCl phase with a relatively positive reduction potential, thereby facilitating charge separation by acting as a sacrificial agent. Consequently, the presence of AgNO_3_ and AO can slightly improve the photocatalytic degradation efficiency of anthracene.

**Fig. 9:**
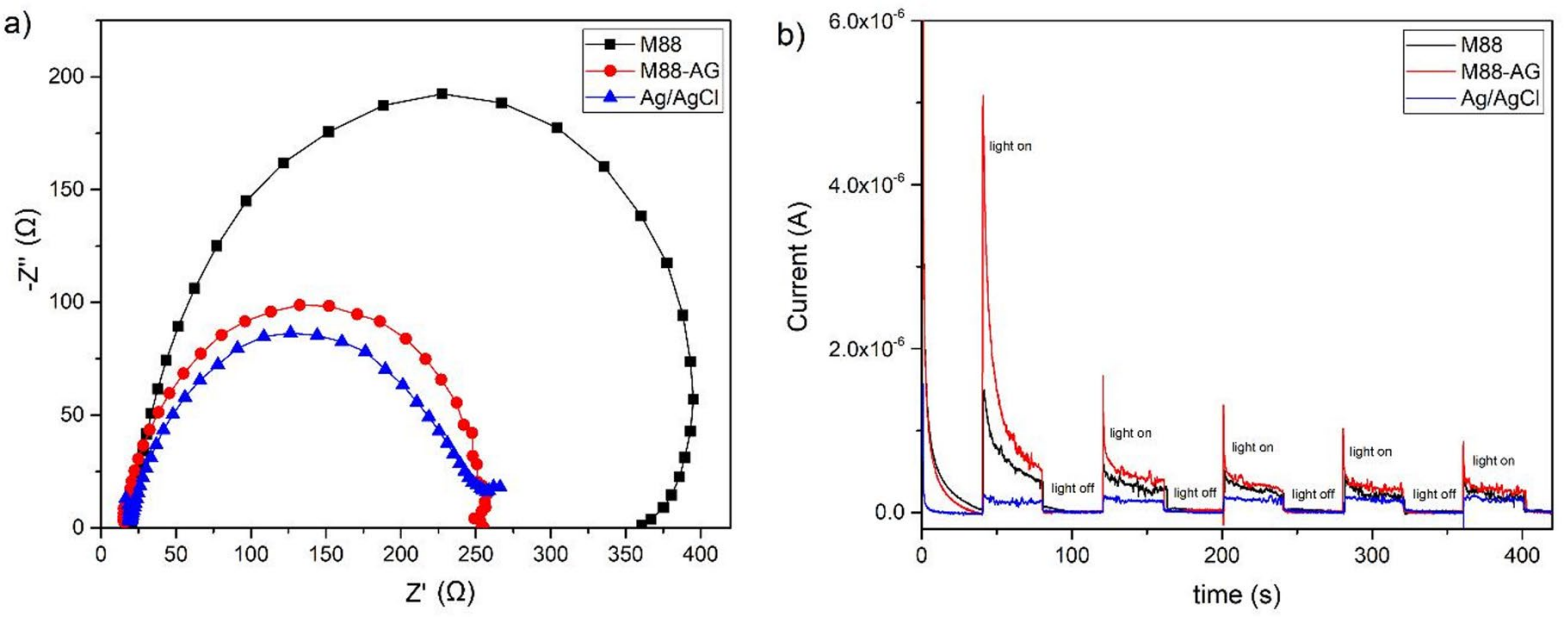
(**a**) EIS Nyquist plots and (**b**) photocurrent of M88, M88-AG and Ag/AgCl.

Electrochemical impedance spectroscopy (EIS) was performed to assess the charge separation efficiency and charge transfer characteristics of the catalysts. The Nyquist plot, which represents the real (Z’) vs. negative imaginary (–Z’’) components of impedance, is shown in Fig. 9a. The diameter of the semicircle in the Nyquist plot corresponds to the charge transfer resistance at the electrode–electrolyte interface, where a smaller semicircle radius indicates lower charge transfer resistance and improved electrical conductivity of the catalyst deposited on fluorine-doped tin oxide (FTO) glass. From the Nyquist plot, it is evident that the M88-AG composite and Ag/AgCl exhibit lower charge transfer resistance compared to pure M88, likely due to the presence of Ag metal, which enhances electron conductivity. This result correlates with the higher photocatalytic activity of M88-AG and Ag/AgCl, as the improved electron mobility facilitates more efficient charge separation during photocatalysis.

To further investigate the impact of heterojunction formation on charge separation efficiency, photocurrent measurements were conducted, as shown in Fig. 9b. Under light irradiation (400–700 nm LED lamp), an increase immediately in photocurrent when light on was observed, indicating enhanced charge carrier generation. Notably, the M88-AG composite exhibited a higher photocurrent response than pure Ag/AgCl, suggesting that the MIL-88B(Fe) component has a band gap energy better aligned with the incident light energy, thereby improving light absorption and charge generation. However, both M88 and M88-AG exhibit a gradual decrease in photocurrent intensity over time. This behavior suggests that the MOF structure may not be sufficiently stable to withstand the applied bias during the electrochemical measurement. Similar photocurrent decay has also been reported for other MOF-based photocatalysts^64^. These electrochemical analyses confirm that the M88-AG composite enhances charge separation efficiency due to heterojunction formation, while the Ag phase improves electron transport within the structure, leading to superior photocatalytic performance.

Gas chromatography–mass spectrometry (GC–MS) was employed to identify the degradation products formed during the photocatalytic oxidation of anthracene and to provide insight into the possible reaction pathways. As shown in Figure S6, the chromatographic analysis revealed anthrone and 9,10-anthraquinone as the major intermediate products. These compounds have been widely reported as primary oxidation products of anthracene in previous studies^14,16^.

**Fig. 10:**
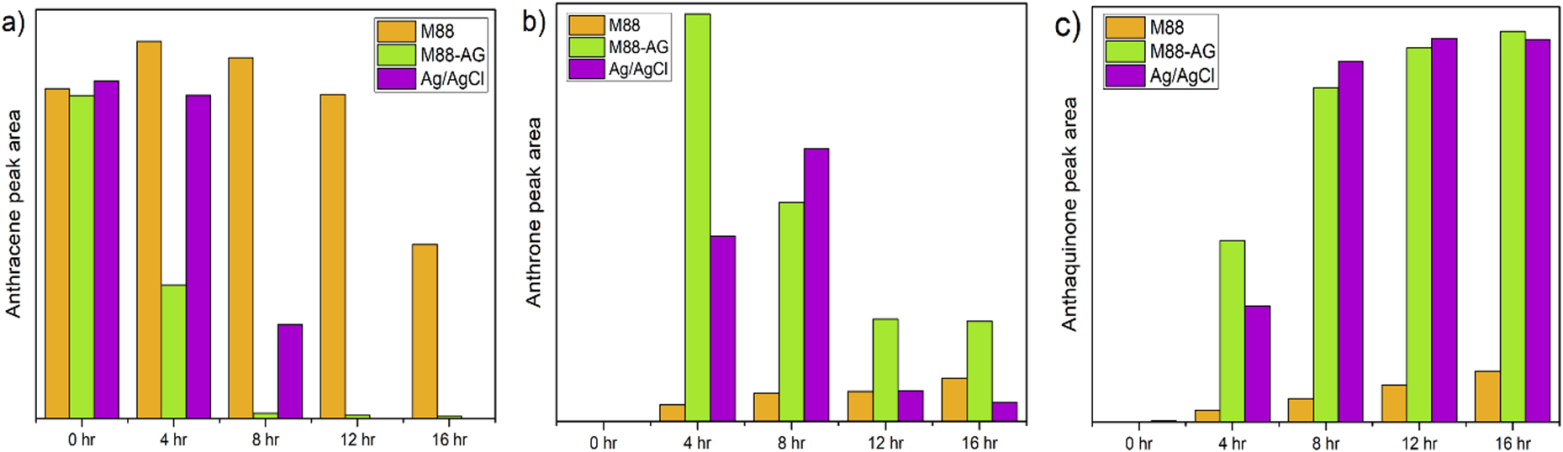
Comparison of GC-MS peak areas for key compounds during photocatalytic anthracene degradation using different catalysts: (**a**) Anthracene, (**b**) Anthrone, and (**c**) 9,10-Anthraquinone.

Figure 10a illustrates the decrease in anthracene concentration over time for the three catalysts tested, with M88-AG exhibiting the highest degradation rate, in agreement with the photocatalytic efficiency results presented in Fig. 7a. A particularly notable intermediate detected in this study is anthrone. As shown in Fig. 10b, the peak area corresponding to anthrone is initially high but gradually declines as the reaction progresses. This suggests that anthrone serves as a transient intermediate^65^, further oxidizing into 9,10-anthraquinone, whose peak area increases over time.

After extended irradiation (16 h), several monocyclic aromatic compounds were detected, indicating further degradation of anthracene-derived intermediates. In the presence of AgCl/Ag, 9,10-anthraquinone was further transformed into phthalic acid, a commonly reported oxidation product of polycyclic aromatic hydrocarbons, consistent with previous studies^62,65^. In contrast, the M88-AG and M88 systems generated different sets of degradation products, suggesting that different degradation pathways may be involved depending on the catalyst composition. Specifically, the M88-AG catalyst yielded methylbenzaldehyde and methylbenzyl alcohol, whereas M88 produced only methylbenzaldehyde. The mass spectra of all identified products are provided in Figure S7.

**Fig. 11:**
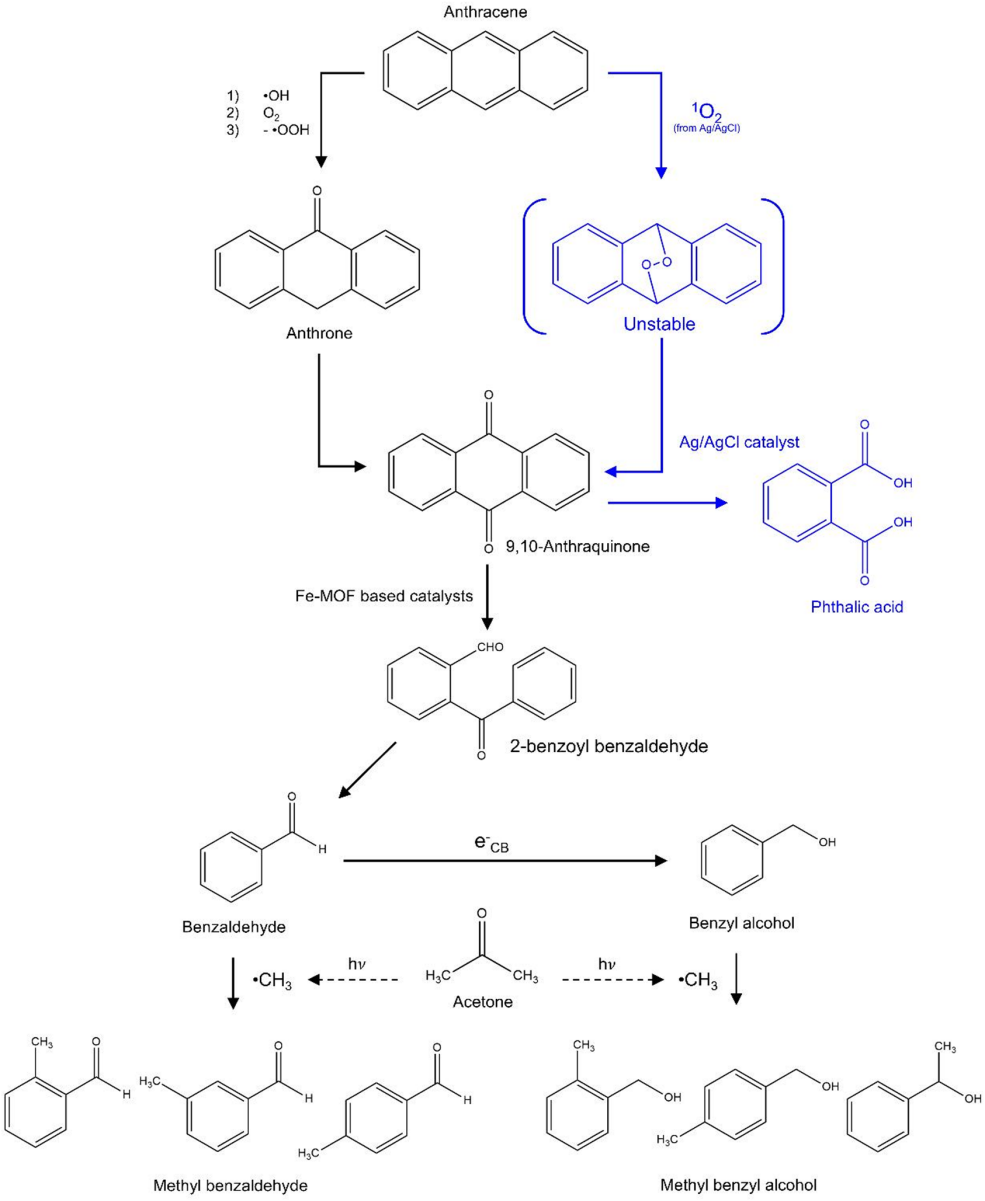
Plausible photocatalytic degradation pathway of anthracene over the MIL-88B(Fe)/AgCl/Ag composite based on the detected intermediates.

Based on the combined results of scavenger experiments, EPR analysis, and GC–MS identification, a proposed pathway for anthracene degradation is illustrated in Fig. 11. Under visible-light irradiation, the MIL-88B(Fe)/AgCl/Ag composite generates reactive species including singlet oxygen (^1^O_2_) from the Ag/AgCl phase and hydroxyl radicals (^•^OH) from the Fe-MOF component. These reactive species initiate the oxidation of anthracene through two primary pathways.

First, ^•^OH radicals attack the central aromatic ring of anthracene, followed by the addition of O_2_ and elimination of a •OOH group, leading to the formation of anthrone as the initial oxidation intermediate. This mechanism has been previously reported with electron-arrow descriptions^65^. Anthrone can subsequently undergo further oxidation to form anthraquinone through a similar radical oxidation process. However, the multiple reaction steps and the relative stability of anthrone may render this pathway comparatively slower.

In parallel, singlet oxygen (^1^O_2_) generated from the Ag/AgCl phase can react with anthracene through a hetero-Diels–Alder reaction, forming an unstable 9,10-endoperoxide intermediate^66^. This intermediate rapidly rearranges or decomposes to produce anthraquinone, which acts as a key intermediate in the degradation pathway. This observation may explain why pure Ag/AgCl exhibits a higher anthracene degradation rate than pristine Fe-MOF (M88), as the singlet oxygen pathway enables faster conversion of anthracene to anthraquinone compared with the radical pathway in the MOF. Meanwhile, the M88-AG composite, which can proceed through both pathways, shows the highest photocatalytic efficiency. In addition, the heterojunction formed between MIL-88B(Fe) and Ag/AgCl enhances charge separation, further improving photocatalytic activity.

To clarify the formation of single-ring aromatic products, repeated photocatalytic degradation experiments were conducted using M88-AG, with additional sampling performed at 10 h. The corresponding chromatograms are presented in Figure S8. In addition to the previously identified products, a signal with a retention time of 15.31 min was observed and assigned to 2-benzoylbenzaldehyde. This compound may serve as an important intermediate leading to the formation of single-ring aromatic products. Under light irradiation, reactive species generated by the composite photocatalyst may induce photocatalytic fragmentation of this intermediate, producing smaller aromatic compounds such as benzaldehyde. Subsequently, benzaldehyde may be reduced by conduction-band electrons to form benzyl alcohol^67^. In the presence of acetone as the solvent, acetone-derived methyl radicals (^•^CH₃) may form under photocatalytic conditions and react with benzaldehyde and benzyl alcohol to produce methylated derivatives such as methylbenzaldehyde and methylbenzyl alcohol. These compounds are difficult to distinguish by mass spectrometry due to their similar fragmentation patterns. Overall, the degradation process involves successive oxidation, photocatalytic fragmentation, and radical reactions that transform anthracene into smaller oxygenated aromatic compounds, which may undergo further oxidation under photocatalytic conditions^68,69^.

## Conclusion

In summary, the photocatalytic performance of MIL-88B(Fe) was significantly enhanced by constructing a ternary MIL-88B(Fe)/AgCl/Ag composite through precipitation followed by photoreduction. The resulting M88-AG photocatalyst exhibited superior anthracene degradation compared with pristine MIL-88B(Fe) and AgCl/Ag under visible-light irradiation. The improved activity is attributed to the formation of an effective heterojunction that facilitates charge separation and promotes the generation of reactive species. Scavenger experiments revealed that singlet oxygen (^1^O_2_), mainly generated from the Ag/AgCl phase, plays a dominant role, while hydroxyl radicals (^•^OH) produced from the Fe-MOF component contribute as secondary reactive species. Mechanistic studies based on UV–Vis and GC–MS analyses suggest that anthracene is first oxidized to anthrone and subsequently to 9,10-anthraquinone, followed by further transformation into lower-molecular-weight aromatic compounds. The Fe-MOF component also facilitates radical-mediated reactions, leading to unique degradation products such as methylbenzaldehyde and benzyl alcohol derivatives. These findings provide insight into anthracene photocatalytic degradation and demonstrate the potential of AgCl/Ag/MIL-88B(Fe) as an efficient visible-light-driven photocatalyst for PAH remediation.

## Supplementary Information

Below is the link to the electronic supplementary material.

Supplementary Material 1

## Data Availability

All data generated or analyzed during this study are included in this published article and its supplementary information file.
