# Supplementary material for: Ultrasonic bath synthesized MIL-88B(Fe)/AgCl/Ag composite for visible-light photocatalytic degradation of anthracene
Source: Sci Rep. 2026 May 6;16:20826. doi: 10.1038/s41598-026-45178-w (PMC13338440; doi:10.1038/s41598-026-45178-w)
Supplement: Supplementary file 1 — Supplementary Material 1 [file 41598_2026_45178_MOESM1_ESM.docx]

**Supplementary Data**

**Ultrasonic Bath Synthesized MIL-88B(Fe)/AgCl/Ag Composite for Visible-Light Photocatalytic Degradation of Anthracene**

Pasu Inphak^a^, Prakasit Intaphong^a^, Sujitra Tandorn^a,c^ and Chamnan Randorn^a,b,^*

^a^Department of Chemistry, Faculty of Science, Chiang Mai University, Chiang Mai, 50200, Thailand

^b^Center of Excellence in Materials Science and Technology, Chiang Mai University, Chiang Mai, 50200, Thailand

^c^Office of Research Administration, Chiang Mai University, Chiang Mai, 50200 Thailand

* Corresponding author.

E-mail address: chamnan.r@cmu.ac.th, crandorn@gmail.com


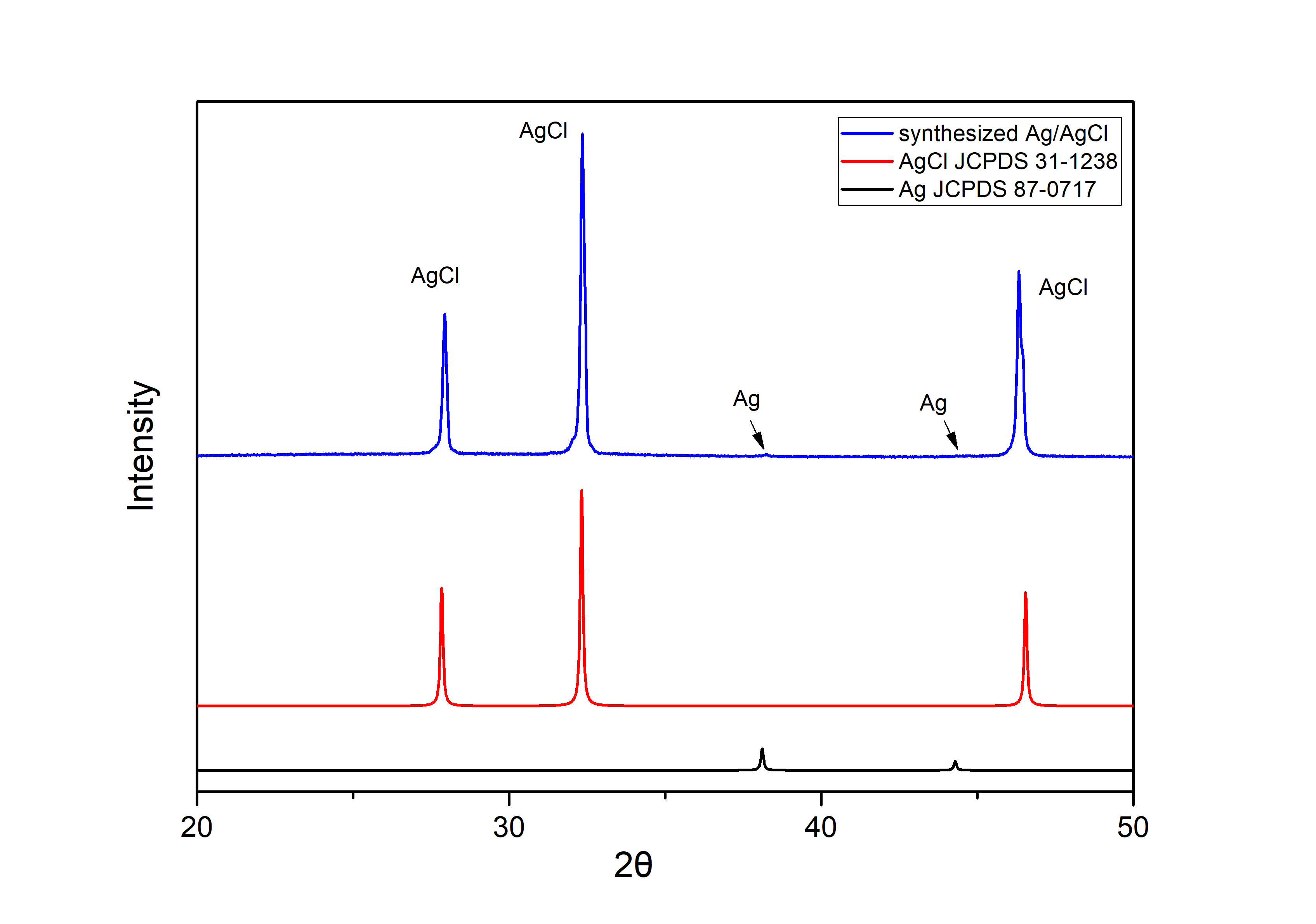


**Figure S1.** XRD pattern of AgCl


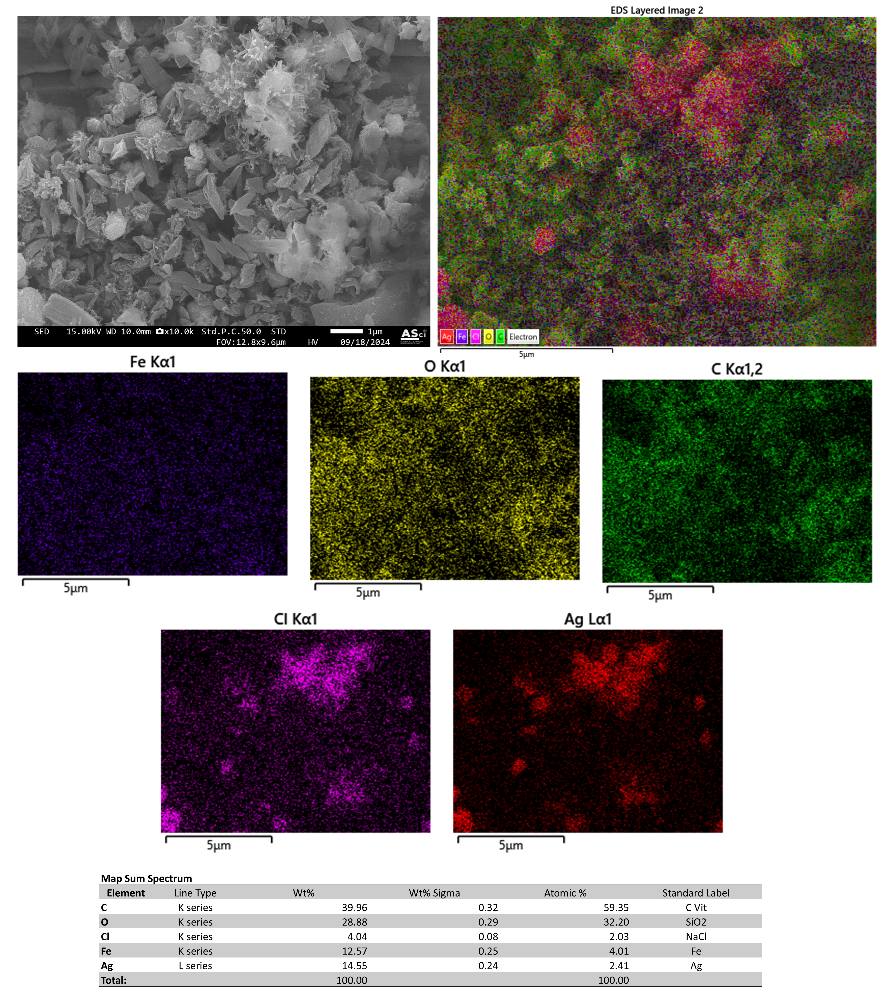


**Figure S2.** EDS-Mapping elemental analysis of M88-AG


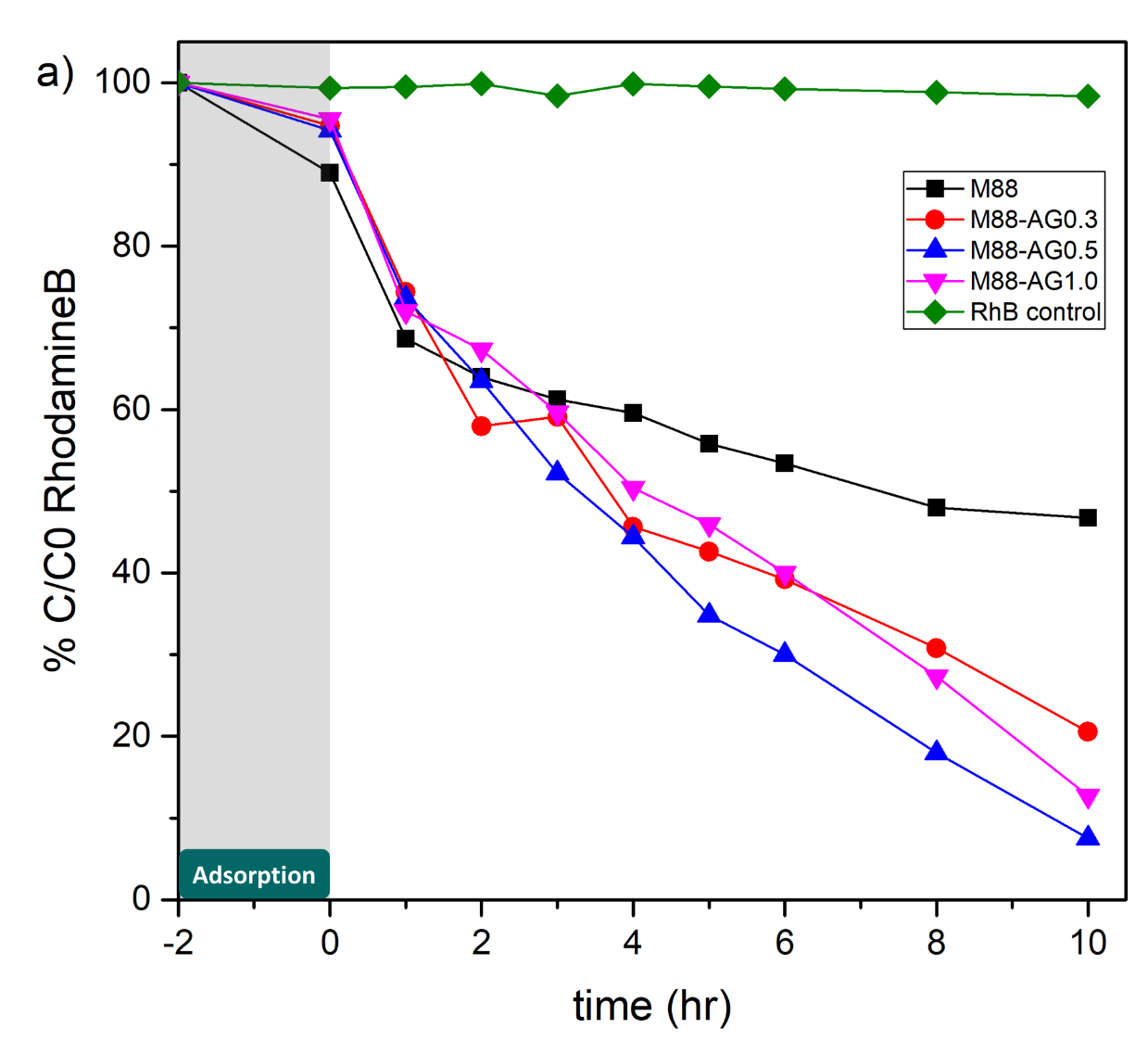


**Figure S3.** comparison of photocatalytic performance in RhB degradation between M88 and M88-AG in various composition


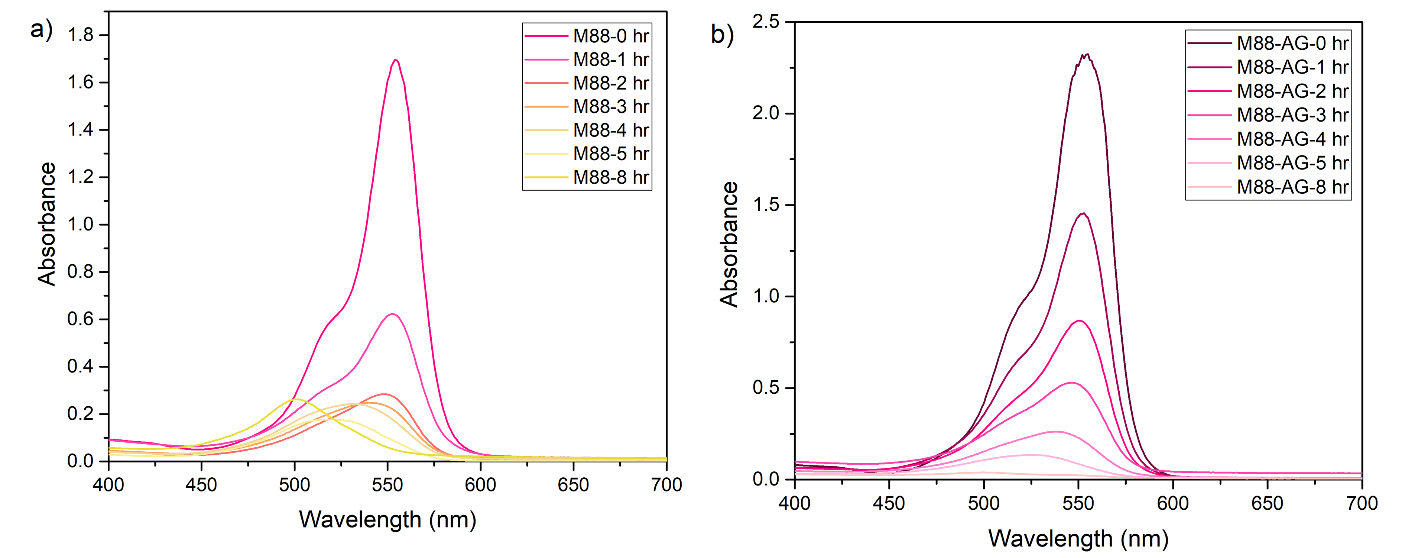


**Figure S4.** UV–Vis spectral analysis of RhB over time of a) M88 and b) M88-AG


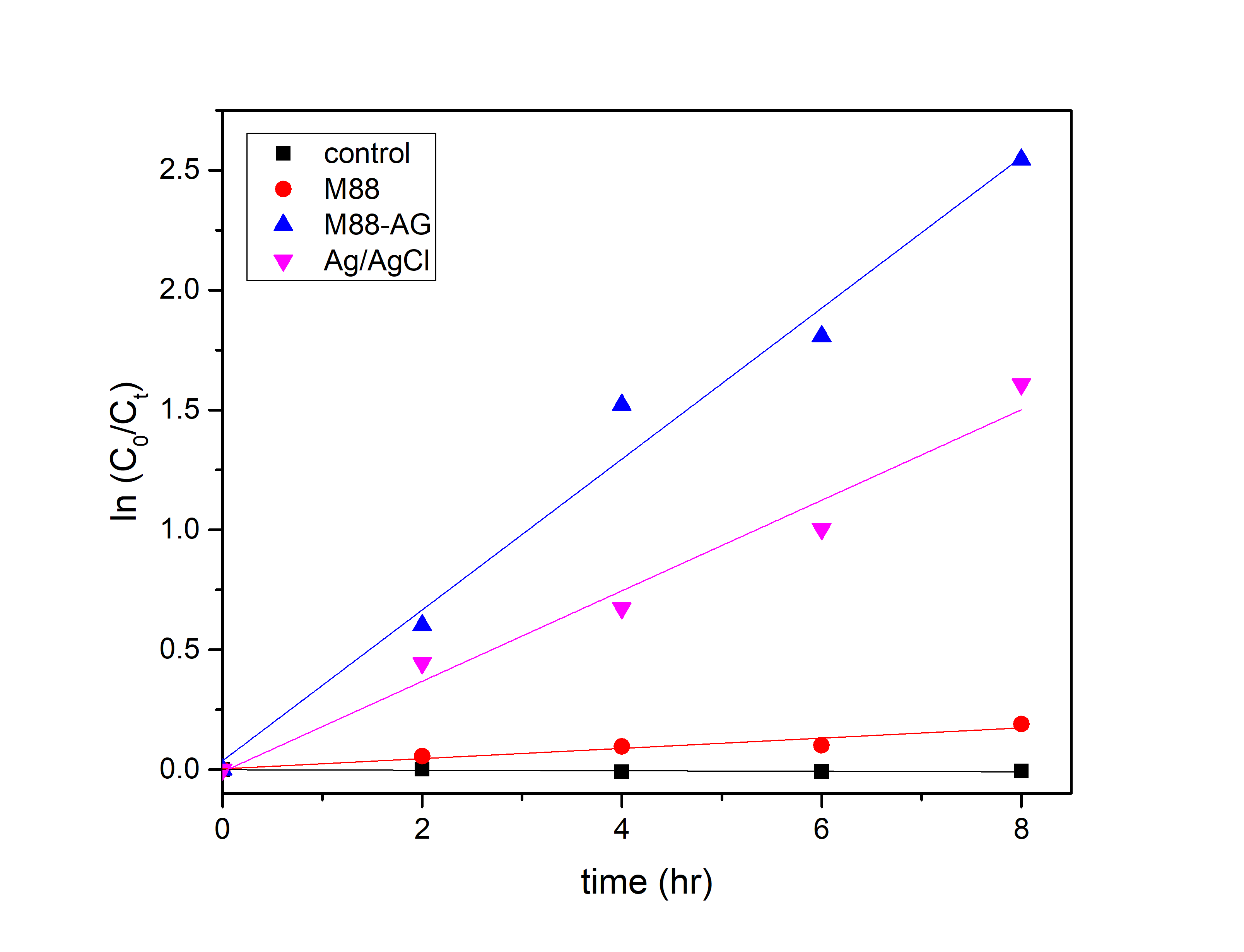


**Figure S5.** The degradation followed first-order kinetics, with calculated half-lives of M88 (32.43 hours), M88-AG (2.20 hours), and Ag/AgCl (3.67 hours)


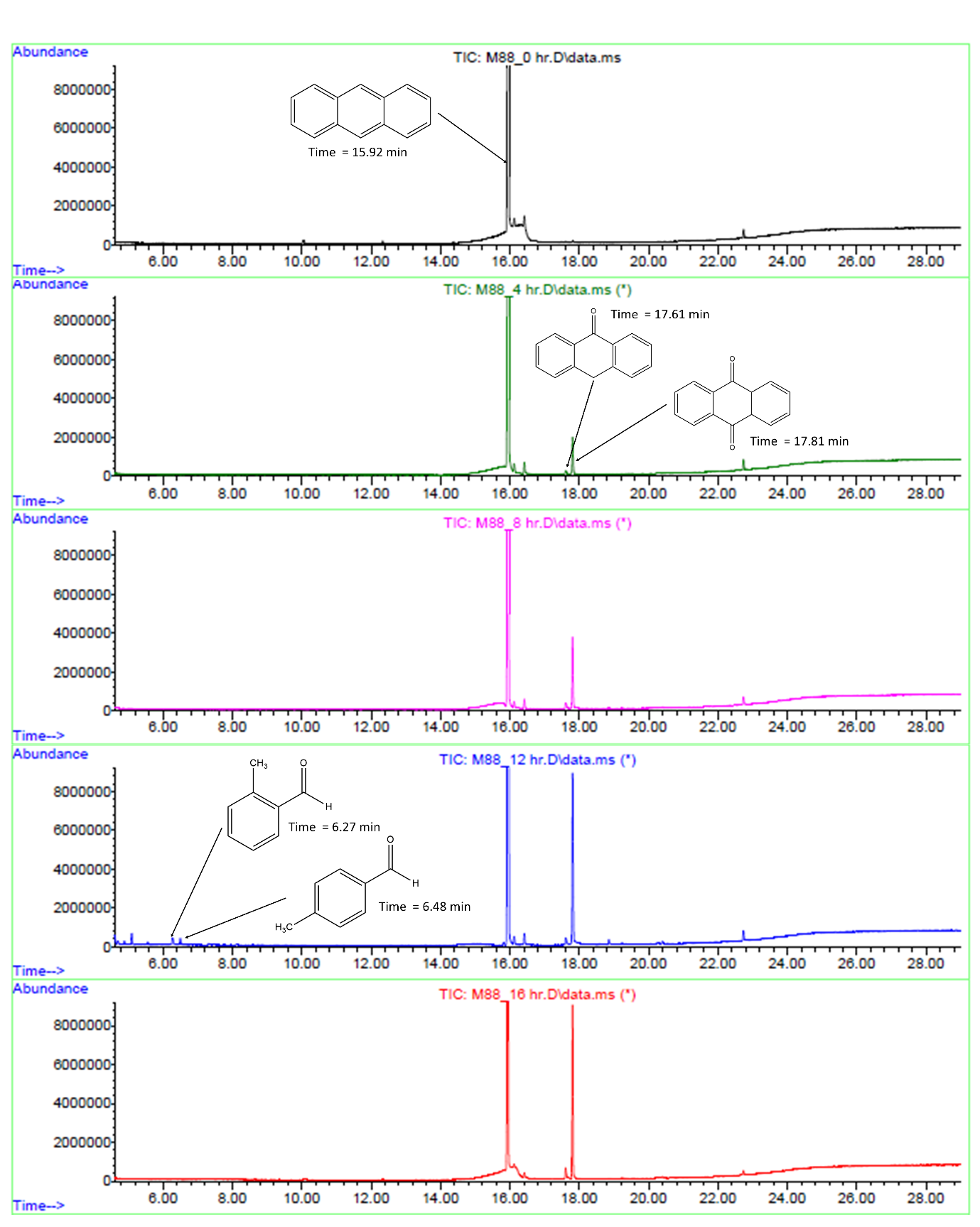


**Figure S6.1.** Gas chromatograms of reaction solutions collected at various time intervals during photocatalytic anthracene degradation using M88.


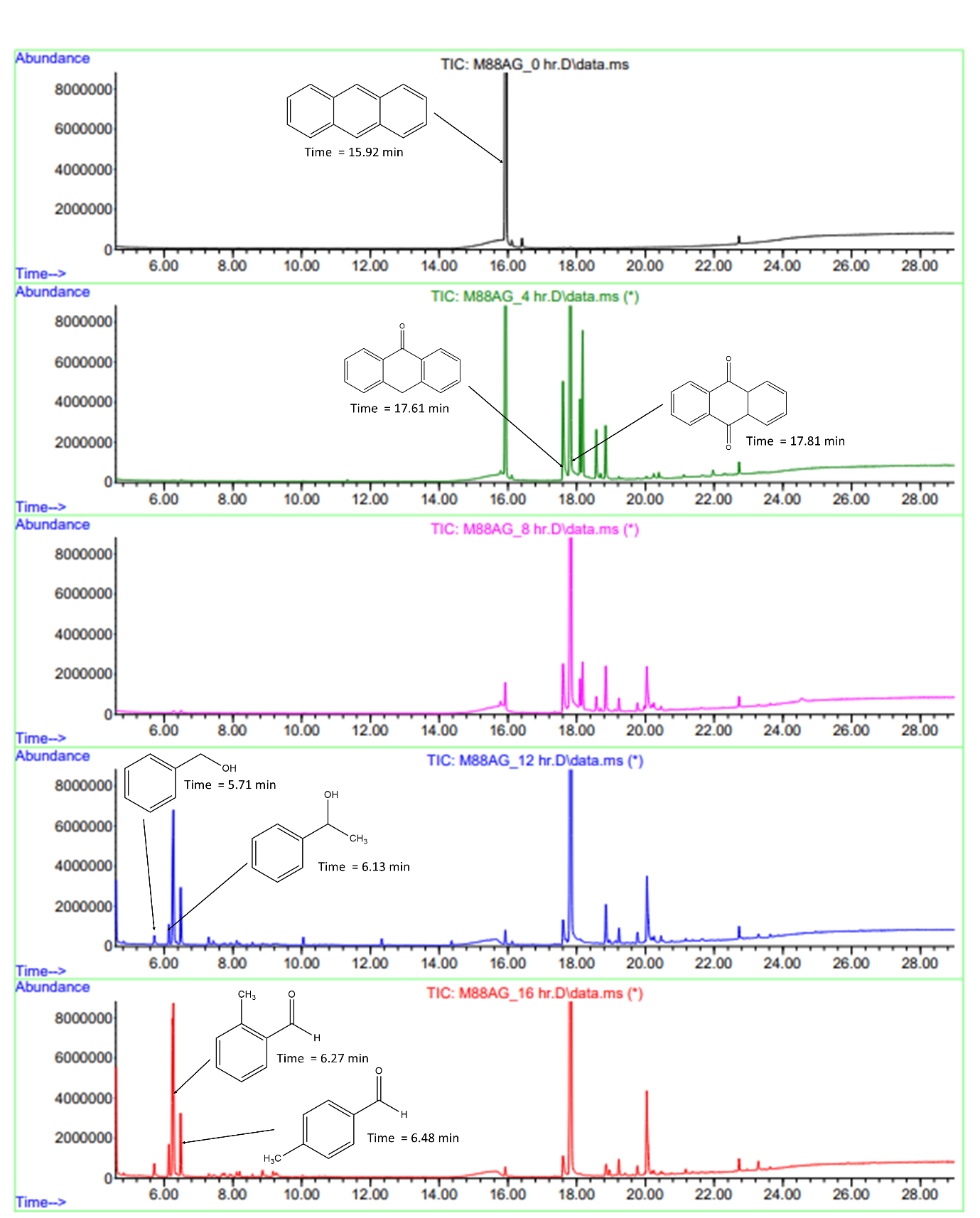


**Figure S6.2.** Gas chromatograms of reaction solutions collected at various time intervals during photocatalytic anthracene degradation using M88-AG.


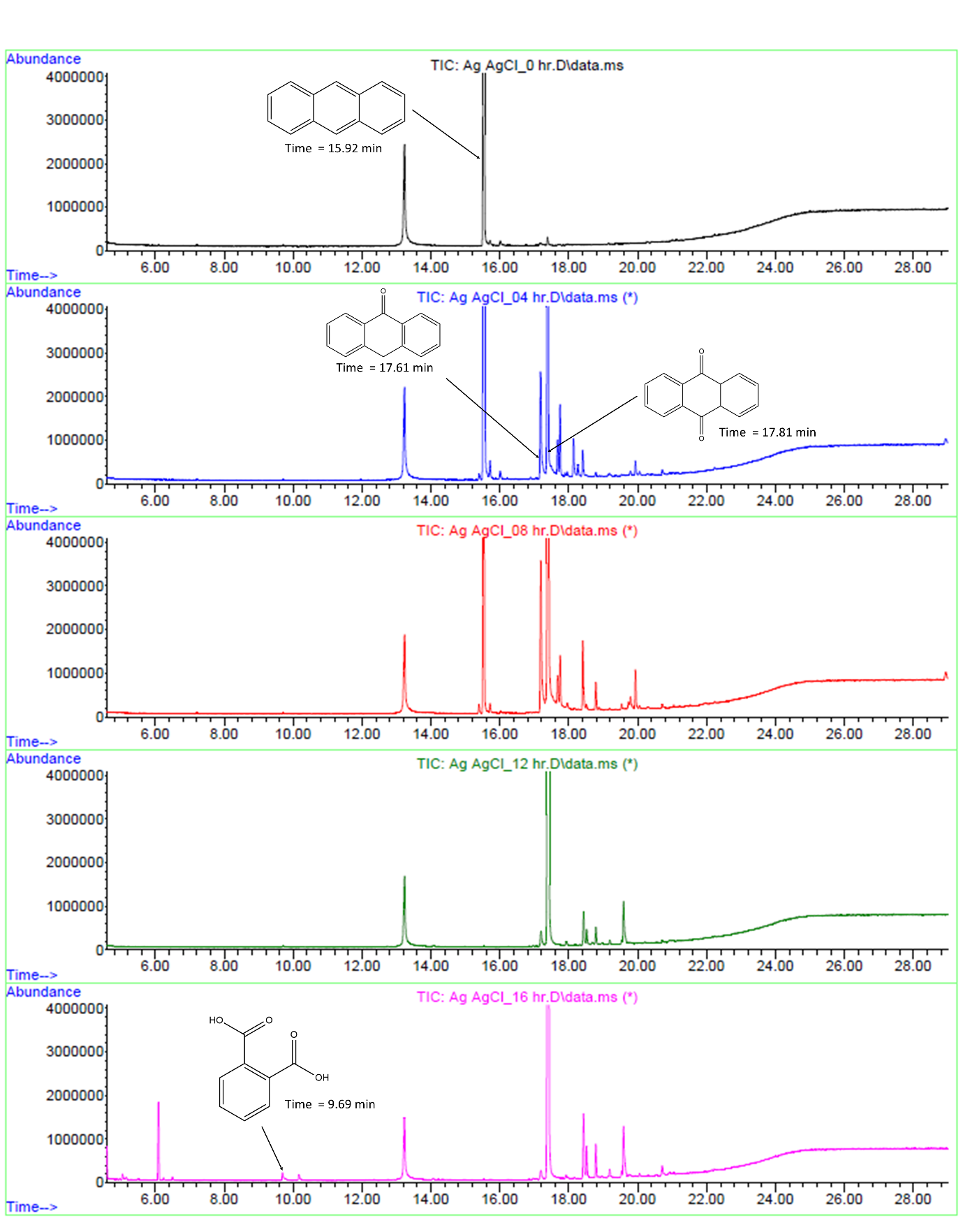

**Figure S6.3.** Gas chromatograms of reaction solutions collected at various time intervals during photocatalytic anthracene degradation using Ag/AgCl.


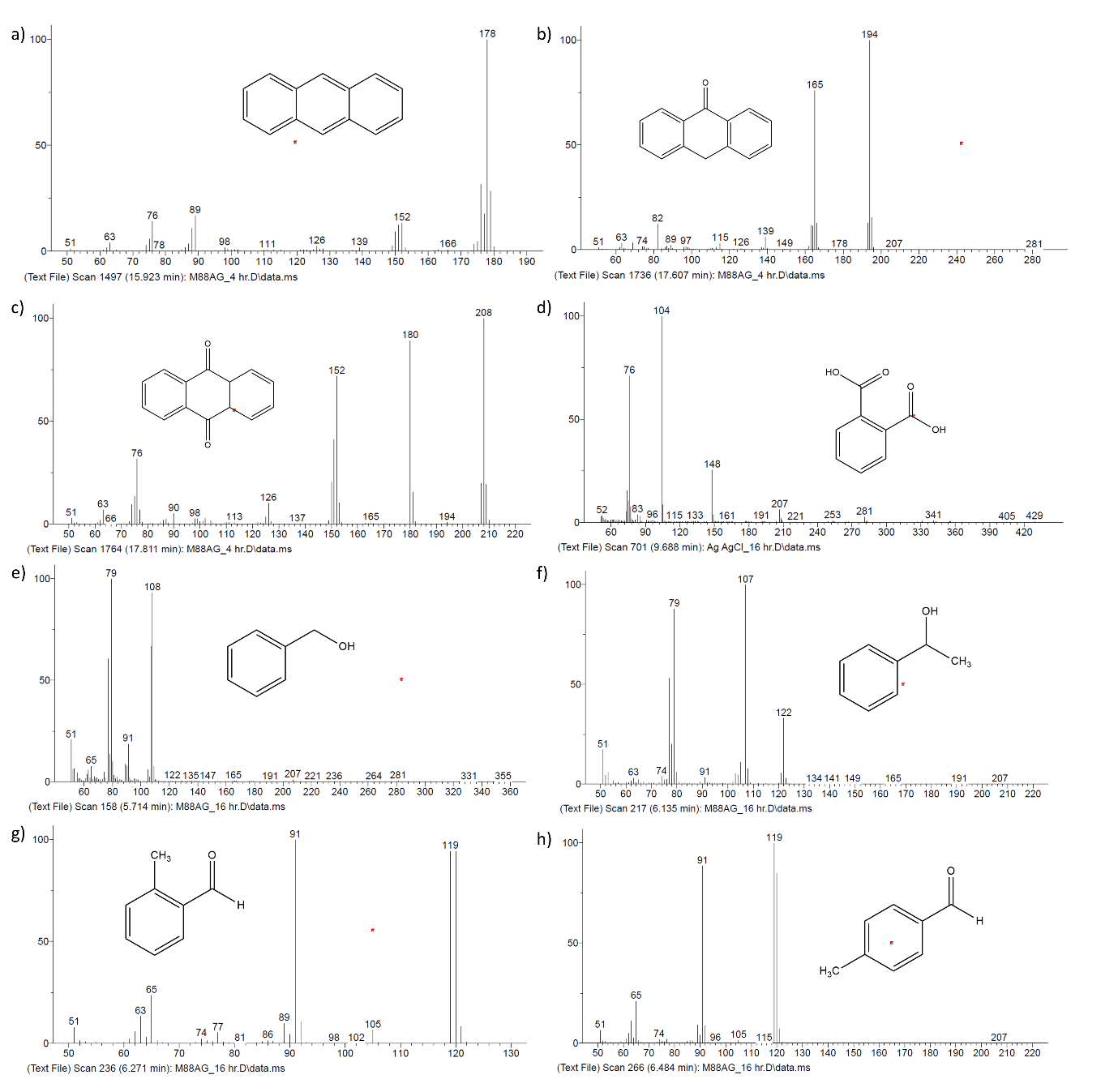


**Figure S7.** Mass spectrum of Anthracene degradation products from GC-MS a) anthracene, b) anthrone, c) anthraquinone, d) phthalic acid, e) benzyl alcohol, f) α-methylbenzenethanol, g) 2-methylbenzaldehyde and h) 4-methylbenzaldehyde

**Table S1.** Summary of peak areas for all identified degradation products from gas chromatograms shown in Figure S6.
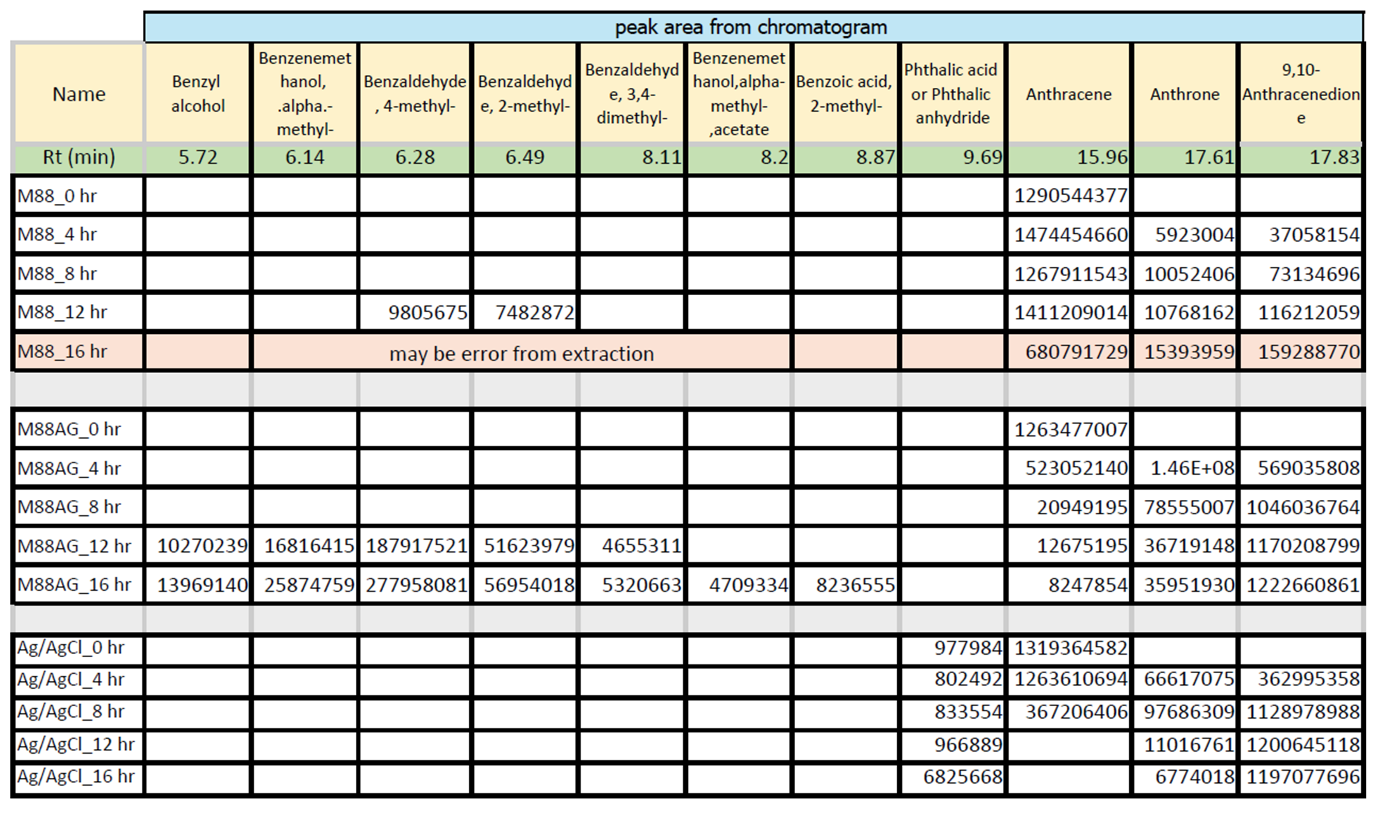


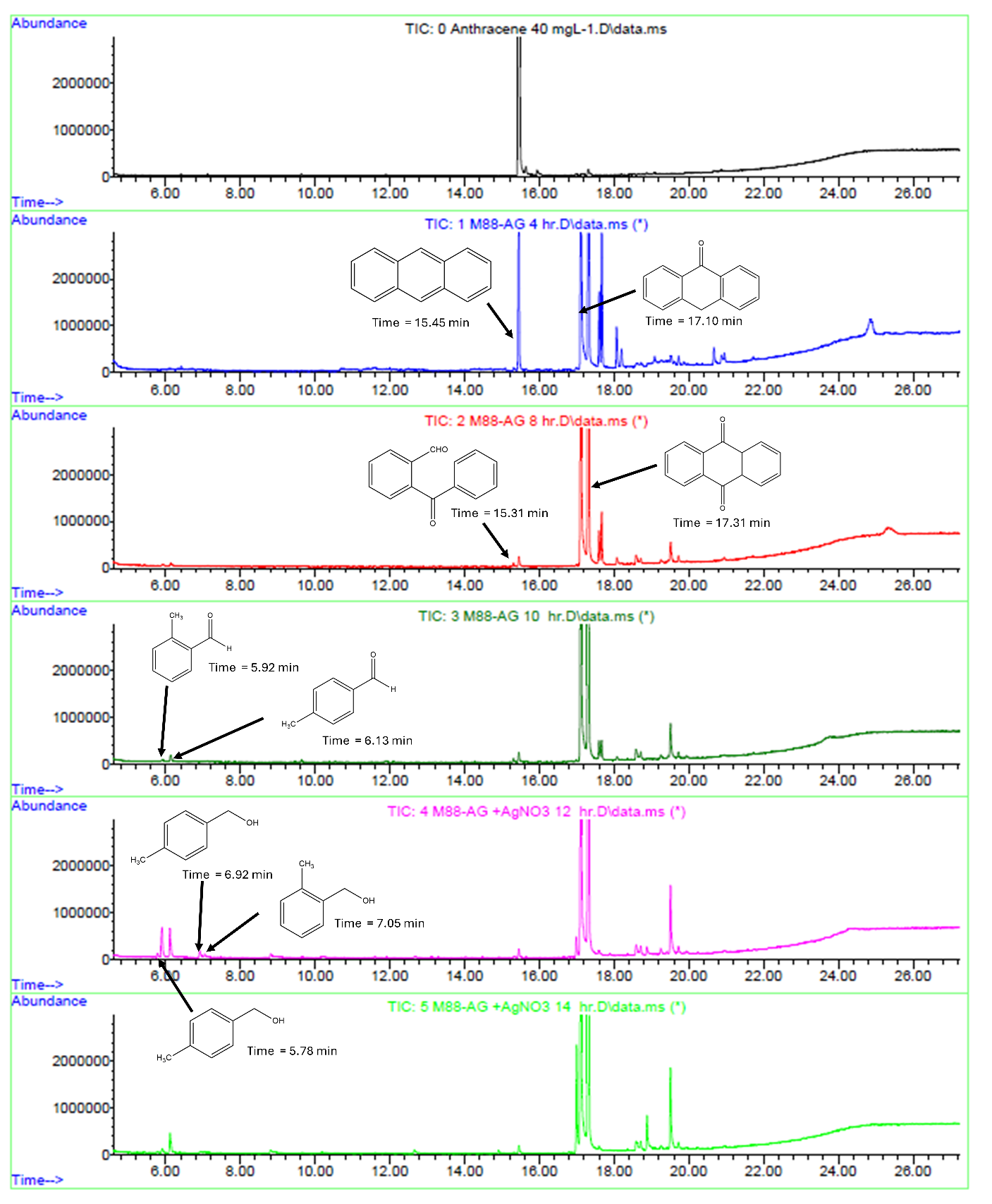


**Figure S8.** Gas chromatograms of reaction solutions collected at various time intervals during photocatalytic anthracene degradation using M88-AG.
